# Process Analytical Technology-Integrated FT-IR and Raman Spectroscopies for Efficient Reactive Liquid–Liquid Extraction Processing in Lithium Recycling

**DOI:** 10.1021/acsomega.5c05705

**Published:** 2025-10-20

**Authors:** Alexander Uhl, Alexandra F. Humann, Axel Schmidt, Jochen Strube

**Affiliations:** Institute for Separation and Process Technology, Clausthal University of Technology, 38678 Clausthal-Zellerfeld, Germany

## Abstract

Lithium is a strategic metal that is essential for the electrification of the economy and society as it is commonly used in high-tech applications and batteries. The EU has mandated that 25% of annual consumption be sourced from recycling. To achieve this goal in an economic and ecological manner, recycling processes need to improve in efficiency. One path toward this aim is by introducing smart control enabled by process analytical technologies (PATs). In this work, a framework for the integration of an in-line spectroscopy system with a chemometric model as a PAT methodological approach for a typical reactive liquid–liquid extraction using a synergistic solvent with a β-diketone is exemplified. The concentration of extractants and the degree of saponification as well as the concentration of metal-ion complexes in the organic phase are to be measured with FT-IR and Raman spectroscopies. This is achieved by generating partial least-squares regression models with a coefficient of regression *R* ^2^ of minimum of 0.95 for application in a continuous process. With these, a reduction of the chemical cost for a typical lithium purification plant of 15% with a reduction in the global warming potential (GWP) of 20% and a return on investment of less than 0.4 years is estimated.

## Graphical abstract

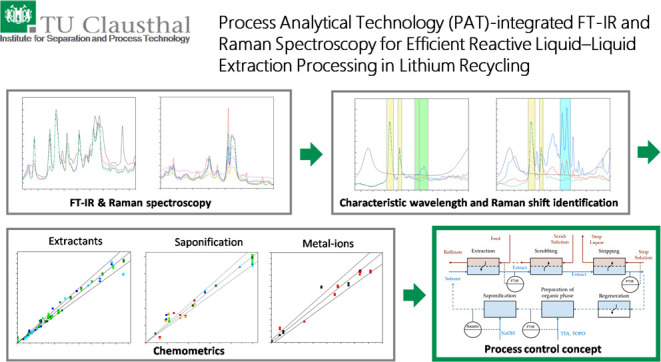

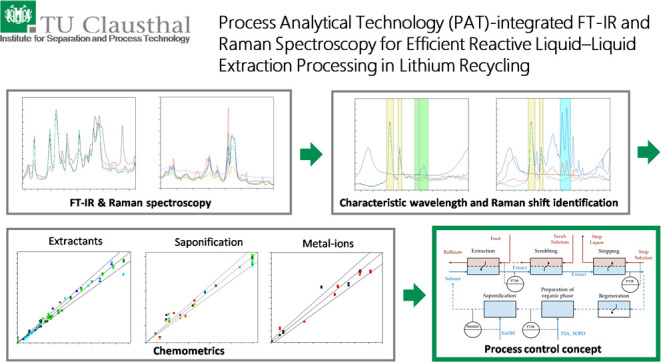

## 1 Introduction

Strategic metals such as rare earth metals, cobalt, nickel, and lithium are highly valued in the current global economy and are therefore also becoming a political issue. To ensure security of supply, i.e., for resilience demands, the European Union has passed the European Critical Raw Materials Act, which sets the goal of diversifying imports of strategic metals. It also stipulates that 25% of the annual consumption of these strategic raw materials should come from domestic recycling by 2030.^1,2^ One particular focus here is on lithium, an alkali metal that is an important component of the high-tech industry, especially lithium-ion batteries (LIBs).^3^

In 2023, approximately 160 kt of LIBs were recycled in Europe,^4^ but these capacities are rapidly increasing, with a capacity of 330 kt/a predicted for 2026.^5^ Therefore, an increase of about 200% must be achieved in an economic and ecological manner.^6–8^

LIBs are manufactured in various compositions and geometries,^9,10^ which makes a uniform recycling process difficult. Common recycling routes for LIBs are either pyrometallurgical or hydrometallurgical. Hydrometallurgical processes achieve higher purity and yield.^11,12^ Pyrometallurgical processing does not require mechanical presorting. However, pyrometallurgical processes often do not recover lithium.^12^ At present, hydrometallurgical processing is still economically difficult when the costs of transport, sorting, and dismantling are considered.^9,10,13,14^

An efficient process needs to be able to control changes effectively; to this end, changes in a continuous process have to be monitored constantly. Process analytical chemistry (PAC) was developed to monitor processes online starting in academia and the chemical industry from 1987, focusing on sensor development and multivariate model development. Process analytical technology (PAT) as introduced in the regulatory field in 2004 focuses on a more holistic view of process development and control.

In batch operations, the process composition and spectral signatures typically evolve throughout each run, so PAC must focus on dynamic calibration sets, transient diagnostics, and robust out-of-calibration detection to capture changes and batch-to-batch variability. In contrast, a continuous process at a fixed probe location is intended to operate in a steady-state window, which simplifies the model design: calibrations can be narrower and centered on the operational envelope, validation can rely on independent steady-state runs, and online monitoring focuses on drift and out-of-specification detection rather than resolving the rapidly changing composition. Continuous processes offer a more cost-effective production and are widely used in chemical and pharmaceutical productions.^15–17^

PAT with the quality-by-design (QbD) framework has a major impact on the efficiency of a process as it can provide previously difficult-to-access information in real time. Moreover, it needs be pointed out that PAT is a methodological approach within QbD, which is therefore no synonym of in-line spectroscopy.^18–20^ Particularly prominent are spectroscopic analysis techniques such as near-infrared (NIR), midinfrared (MIR), Fourier transform infrared (FT-IR), and Raman spectroscopies, which can be quickly evaluated using chemometric analyses and provide conclusions about concentrations and purity in an ongoing process.^21–26^ This opens up new control options that can respond directly to the quality attributes of the product like concentrations and purities instead of process indicators such as pressure or temperature. A more efficient manufacturing process is achieved, meaning it uses fewer chemicals, emits less CO_2_, and keeps the cost of the product lower. It also allows a response to varying compositions of the feed stream, keeping product quality in specification. Model predictive control supported by digital twins can also be used for this purpose. A digital twin is by definition an experimentally validated process model which gets a feedback of the existing actual process operation status from sensors and adapts in order to mirror this state exactly in order to be able to give feedback actions back to the process.^27–29^ With the addition of in-line spectroscopy, it becomes possible to dispense with time-consuming off-line analysis and to quickly check and approve products for their specifications.^30–32^ Implementation cost and maintenance have to be evaluated as demonstrated later individually. The need for process monitoring in hydrometallurgical processing using fast and accurate process analysis technologies has been discussed as early as in 1975, and proposals for measuring extraction agents using X-ray fluorescence (XRF) were demonstrated experimentally.^33^ The composition of feedstock from recycled materials vary more than from primary resources,^9,10^ and more accurate information is required for controlling a process. Therefore, PAT is particularly applicable to recycling processes. For the implication of PAT, optimal sensors and models must be developed.

In this work, FT-IR and Raman spectroscopy are investigated for PAC process monitoring in a continuous lithium purification process using a synergistic β-diketone system. Three applications of PAC have potential: concentrations of the extraction agents, the degree of saponification of the organic phase, and the concentrations of metal-ion complexes in the organic phase. Therefore, three series of solutions are prepared, which are measured with FT-IR and Raman devices. These cover the expected operating window for possible steady states of the continuous process, thereby enabling process control. For each application characteristic, wavenumbers or Raman shifts are identified by academic literature from the chemical structure, and the spectra are preprocessed. The resulting spectra are used to create and validate partial least-squares (PLS) and multivariate curve resolution-alternating least-squares (MCR-ALS) regression models. On this basis FT-IR and Raman spectroscopies are compared as PAC sensors for different applications in a typical hydrometallurgy process. Subsequently, the possible use in a QbD-based process as PAT application of this PAC and its optimization potential for solvent extraction are discussed and evaluated.

## 2 State of the Art

Liquid–liquid extraction for metal-ion purification is a subfield of hydrometallurgy, in which, especially reactive liquid–liquid extraction, or often named shortly solvent extraction, is a prominent unit operation. In this process, ligands in the organic phase complex metal ions from the aqueous phase. In most cases, these reactions are dependent on the pH value. Oxygen-based chelators such as crown ethers, β-diketones, and phosphorus-based acidic extractants or nitrogen-based chelators such as macrocyclic aza-crown ethers or phosphorus- and sulfur-based chelators are known for the extraction of lithium.^34^

A typical flow diagram for continuous solvent extraction is shown in Figure 1. The operation is divided into four steps: extraction, scrubbing, stripping, and regeneration. During the extraction, as many of the target metal ions as possible are transferred from the aqueous phase to the organic phase. This is then purified in the scrubbing step with the aim of selectively removing the secondary components from the organic phase. In the stripping step, the target metal is extracted from the organic phase, which is then regenerated and recycled in the next extraction. Each step can consist of several stages, and the pH value and phase ratio can be changed in each step to achieve the purification goal.^35^

**Figure 1:**
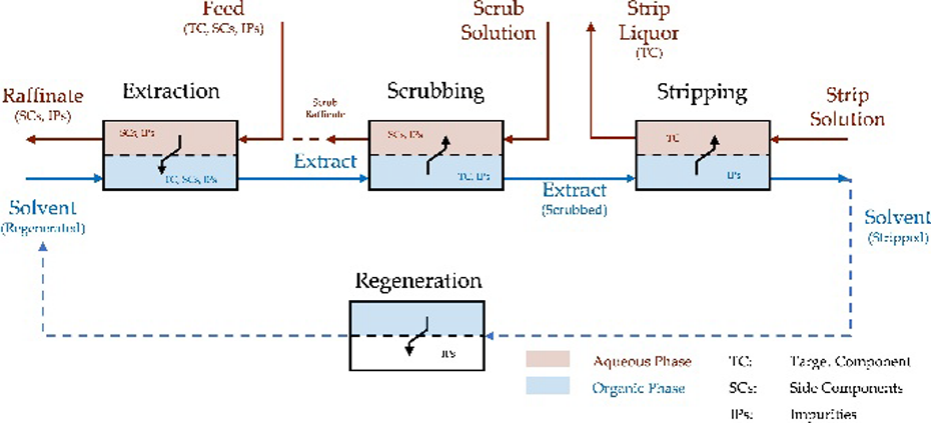
General solvent extraction loop for separation of metal ions.^35,36^ Adapted with permission from Schmidt and Strube, 2018.

For this work, a system with the extraction agents 1-(2-thenoyl)-3,3,3-trifluoroacetone (TTA) and trioctylphosphine oxide (TOPO) is selected. TTA is a β-diketone that, together with TOPO, can complex a lithium ion in the organic phase. This system has already been characterized in the literature in terms of the influences of the concentration of the ligands and the pH values required for extraction. A correlation can be observed between the concentration of TOPO and the extraction efficiency at a constant concentration of TTA in the organic phase. An optimum was observed at a molar ratio of TTA to TOPO of 1:2. From these analyses, it is known that the complexation of TTA and TOPO with a lithium ion proceeds according to the following reaction scheme^37,38^ and is shown in Figure 2.

$${\mathrm{Li}}^{+}+{\mathrm{OH}}^{-}+\mathrm{TTA}+2\mathrm{TOPO}⇌H_{2}O+[\mathrm{Li}(\mathrm{TTA})(\mathrm{TOPO}{)}_{2}]$$

**Figure 2:**
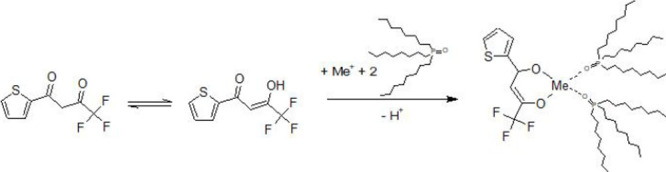
Reaction scheme of the formation of a metal-ion complex.

The equilibrium pH value is the most important parameter for extraction efficiency in the extraction agents used in the literature, such as Cyanex 272, Cyanex 301, and D2EHPA.^11^ This is also the case with the extraction agents TTA and TOPO used here, where a pH value of up to 10, depending on the concentration of lithium and the extraction agent, is required for maximum extraction efficiency.^9,37,39^ This also influences the separation factor of lithium from other metals, such as sodium.^40^ Similarly, the efficiency of stripping the organic phase is dependent on the equilibrium pH value. Harvianto and Zhang both find a strong correlation between the equilibrium pH value or the concentration of the acid used and the efficiency of stripping.^39^ Chemically similar ligands in benzoyl-1,1,1-trifluotoacetone (HBTA) and 4,4,4-trifluoro-1-furoyl-1,3-butanedione with TOPO showed similar dependencies on the pH value and corresponding process conditions.^41,42^

Other applications of the TTA/TOPO system that do not involve classical solvent extraction are known. The TTA/TOPO system is highly effective as an ion acceptor for the separation of Li and Na or K with a cellulose triacetate membrane separator, with a separation factor of over 50 calculated in each case.^43^ In addition, TTA/TOPO has also been investigated as a deep eutectic solvent in which no solvent is used. It can be observed that a lower pH value is required for the same extraction efficiency when comparing the solutions of TTA and TOPO in toluene.^40^

### 2.1 PAT in Hydrometallurgy

The state of the art is the off-line analysis of lithium and other metals using atomic absorption spectroscopy (AAS), inductively coupled plasma optical emission spectroscopy, and inductively coupled plasma mass spectrometry.^44^ These analytical techniques are highly precise and accurate but require complex sample preparation and are suitable for online measurement only to a limited extent.

X-ray diffraction and XRF have been researched both as off-line analysis and in online applications.^6,45^ XRF is particularly suitable for solids or slurries.^46–48^

Single metal ions are not detectable by FT-IR or Raman due to the fact that ions have by definition no covalent bonds that absorb light. Therefore, only metal-ion complexes can be observed through spectroscopic analysis. FT-IR has been used extensively in a qualitative manner to explain molecule and complex structures in hydrometallurgy.^43,49–53^

NIR and Raman spectroscopies have already been developed as PAT for plutonium uranium reduction extraction (PUREX). Nitric acid is an important process parameter in PUREX.^54^ To analyze this concentration online, a PLS model using a deconvoluted Raman spectrum is calibrated and validated. The concentrations of nitric acid and hydrochloric acid as well as the ionic strength and temperature were varied to obtain a robust model.^54^ In addition, the same working group developed a micro-Raman probe with a sample size of 10 μL. This can provide a sufficiently good prediction of the concentrations of NO^3–^ and UO^2–^. This simplifies its use for online and off-line analyses.^55^ Nee et al. achieved very good results for NO3^–^, H^+^, ND3^+^, and Na^+^ from a combination of Raman, NIR, and conductivity measurements in aqueous solution.^56^ Nitric acid prediction with MIR and Raman was also performed in the presence of uranium, as in real process solutions, in the organic phase.^57^

In addition, in-line Raman measurement was used to determine the kinetic mass-transfer coefficient in a two-phase system of nitric acid in tributyl phosphate and vice versa.^58^ An integrated system for extraction with centrifugal extractors for PUREX with an aqueous feed of neodymium nitrate and nitric acid in contact with an organic phase of TBP/n-dodecane was demonstrated.^59^

### 2.2 Multivariate Data Regression

PLS regression is a multivariate calibration technique that reduces large, collinear spectral data sets to a small set of predictive factors, each chosen to maximize the relationship between the measured spectra and known analyte concentrations. This approach is highly robust to noise and overlapping signals, making it a standard tool for quantitative analysis and process monitoring in spectroscopic applications.^60^

MCR-ALS is a self-modeling method that resolves complex spectral data into pure spectra of individual components and their concentration profiles over time, rather than fitting predefined targets as in PLS.^61^

By applying simple chemical constraints, such as non-negativity of spectra, non-negative concentrations, and initializing MCR-ALS with the concentrations measured, regressions are formed. MCR-ALS identifies how many species are present, when they appear or disappear, and what their spectra look like, offering direct insight into evolving processes. Common uses include the resolution of chemical structure from complex spectra.^62^ Like PLS, MCR-ALS is applied in PAT applications.^63^

## 3 Materials and Methods

### 3.1 Analytical Techniques

#### 3.1.1 FT-IR

FT-IR analysis is performed with a ReactIR 702L spectrometer from Mettler Toledo (Greifensee, Switzerland). For the measurements, a DiComp Diamond probe is used. The resolution is set to 4 cm^–1^ for all measurements with a measuring range from 3000 to 650 cm^–1^. FT-IR spectroscopy is performed at room temperature.

#### 3.1.2 Raman Analysis

The Raman instrument Kaiser Analyzer RNX2 (Rnx 785 HPG Multichannel) from Kaiser Optical Systems (Endress+Hauser Group Services AG, Reinach, Switzerland) is used to analyze mostly organic samples. The measurements are carried out by a Kaiser Optical sys SARL probe with a resolution of 4 cm^–1^ and a measuring range between 100 and 3425 cm^–1^ using a 785 nm laser. The length of a single measurement is modified depending on the sample itself. Raman spectroscopy is performed at room temperature.

#### 3.1.3 AAS

Aqueous samples containing metal ions are analyzed with a Varian AA140 (Agilent Technologies Inc., Santa Clara, CA, USA) instrument. The instrument is run with an acetylene flame and calibrated using two standard solutions. For sodium, the standard solution is bought from VWR International (Leuven, Belgium), while the lithium standard is purchased from Merck KGaA/Supleco (Darmstadt, Germany).

### 3.2 Organic Phase Preparation

The organic phase used during this study consists of TTA and TOPO dissolved in kerosene. TTA is purchased from Sigma-Aldrich (Saint Louis, MO, USA) with a purity of 99%, while TOPO is bought from ThermoFischer Scientific (Kandel, Germany) with a purity of 90%. The kerosene (Sigma-Aldrich) provides a purity of reagent grade and is classified for usage in analytical testing. The standard composition of the organic phase for most samples is TTA:TOPO in a ratio of 1:2 with a total weight fraction of 30 wt %.

For the identification of pure TTA and TOPO and their mixtures in kerosene, several sample sets are produced. In two separate sample sets, the quantifiability of TTA and TOPO in kerosene at different concentrations (ranging from 3 wt % up to 30 wt %) was examined. Additionally, samples with different ratios of TTA and TOPO (1:1, 1:2, and 1:3) and different total mass fractions (ranking from 20 to 30 wt %) are produced. The organic compounds are dissolved under strong stirring at room temperature. All samples are analyzed with Raman and FT-IR.

### 3.3 Degree of Saponification

The calculations of the degree of saponification are based on the initial concentrations of TTA and free hydroxide ions in the solution. During the saponification, TTA has to be present in its enol form and will be deprotonated by the present hydroxide ions. The deprotonation mechanism is visualized in Figure 3. Based on these two concentrations, the degree of saponification is then calculated by eq 1.^7^

$$\mathrm{DS}=\frac{c_{0}({\mathrm{OH}}^{-})}{c_{0}(\mathrm{TT}A)}$$ 1

**Figure 3:**
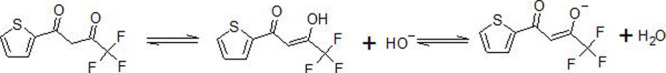
Reaction scheme of the saponification of TTA.

For the determination of the degree of saponification, samples with three different bases are prepared. The samples containing sodium hydroxide (Merck KGaA, Supelco, Darmstadt, Germany) or lithium hydroxide (Sigma-Aldrich) are prepared in the same way, assuming full dissociation of NaOH and LiOH in the organic phase. For the third saponifier, ammonia (Merck KGaA, Supleco), partial dissociation in the aqueous and organic phases is assumed. It is stated that the initial concentrations of ammonium and hydroxide ions are constant, and a complete phase transition of hydroxide ions into the organic phase occurs. The concentration range of all bases is chosen to reach a theoretical degree of saponification between 0 and 1 (leading to a total deprotonation of TTA).

### 3.4 Extraction and Stripping

A selection of the organic phases, loaded either with NaOH or LiOH, is extracted with water. Aqueous:organic-ratios from 1:1 up to 1:4 are chosen for these experiments. The extraction followed the same procedure as saponification with ammonia solution. The resulting organic phases are analyzed with Raman and FT-IR, while only FT-IR measurements for the aqueous phases are performed. Furthermore, the pH values of the aqueous phases are determined.

The loaded organic phases (containing either NaOH or LiOH) are stripped by using two different stripping patterns. The sample sets for lithium and sodium are treated separately but following the same procedure. The sample sets are divided into two groups. The first group contained all samples that are already extracted with water. These samples are first stripped with sulfuric acid (VWR International S.A.S., Fontenay-Sous-Bois, France) at a pH of 3. Afterward, they are stripped again with sulfuric acid at a pH of 1. The second sample group is stripped in both steps with a sulfuric acid solution at pH 1. The aqueous solutions are analyzed with AAS and partially with FT-IR, while the organic phases are analyzed with FT-IR and partially with Raman.

### 3.5 Solubility Testing

To better understand the behavior of TTA and TOPO in aqueous solutions under different conditions, a brief testing of their solubility is performed. Several alkaline and acidic solutions are prepared containing either TTA or TOPO. The aqueous solutions are filtered and then measured with Raman and FT-IR.

### 3.6 Data Preprocessing and Model Formulation

The preprocessing of the obtained spectra is based on statistical analysis. Savitzy–Golay derivates and standard normal variate (SNV) are used in the preprocessing steps. In some cases, additional transformation methods, such as baseline correction, are performed. With the help of the principal component analysis (PCA), outliers in the spectral data set are identified and excluded from further analysis. All of the shown spectra are raw spectra without any preprocessing. To compare the effects of the preprocessing on the spectral data, the final processed spectra are presented in the Supporting Information.

For the PLS regression model development, the obtained data set is randomly split into three parts. 50% of the data was used as a training set, 25% was used as a validation set, and the remaining 25% was used as an external test set. A nonlinear iterative partial least-squares algorithm is employed with a maximum of five orthogonal factors to limit overfitting. The optimal number of latent factors is determined as the minimal number of latent factors with a sufficient validation root-mean-squared error (RMSE) and *R* ^2^. To avoid artifacts of the validation, the RMSE and *R* ^2^ of training and validation are required to be similar.

## 4 Results

### 4.1 Quantification of TTA and TOPO in Kerosene

The detection of TTA and TOPO in the organic phase was performed to achieve two different goals. The first one was the identification of TTA and TOPO.

Figure 4 presents the resulting FT-IR spectra of the analysis. In both spectra, kerosene shows two significant bands in the wavenumber range between 1500 and 1300 cm^–1^. These bands are assigned to the deformation vibrations of the hydrocarbon chains.^64^ Especially in the TOPO spectrum, the kerosene signals show high intensities compared to the TOPO signals. This is caused by the fact that TOPO and kerosene contain long hydrocarbon chains. Both spectra show an increase in the number of significant bands correlated with an increase in the concentration of either TTA or TOPO. For TTA, several significant bands could be detected in the FT-IR spectrum. The most prominent band is identified in the range of 1200 cm^–1^, which is assigned to the stretching vibration of the C–F bonds.^64,65^ The vibrations of P–O and P–C are identified in two regions, close to each other. The symmetric deformation vibration of the P–C bond is located in the higher energy range compared to the P–C bond.^64,65^

**Figure 4:**
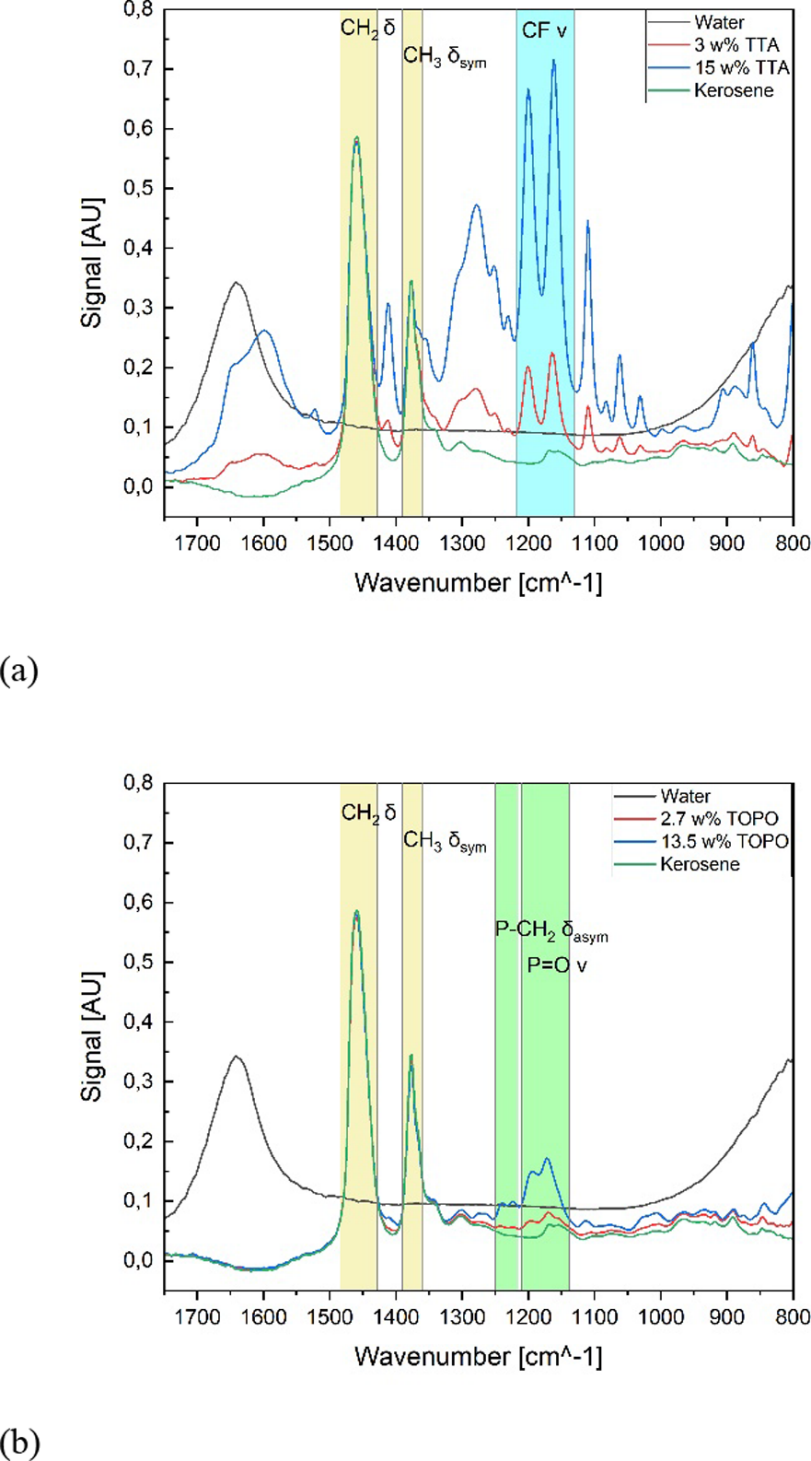
Raw FT-IR spectra of (a) TTA in kerosene and (b) TOPO in kerosene.

The Raman spectra of TTA [see Figure 5a] and TOPO [see Figure 5b] prove similarities compared to the FT-IR spectra. It can be seen that the intensity of bands, corresponding either to TTA or TOPO, is increasing with an increase in their concentrations.

**Figure 5:**
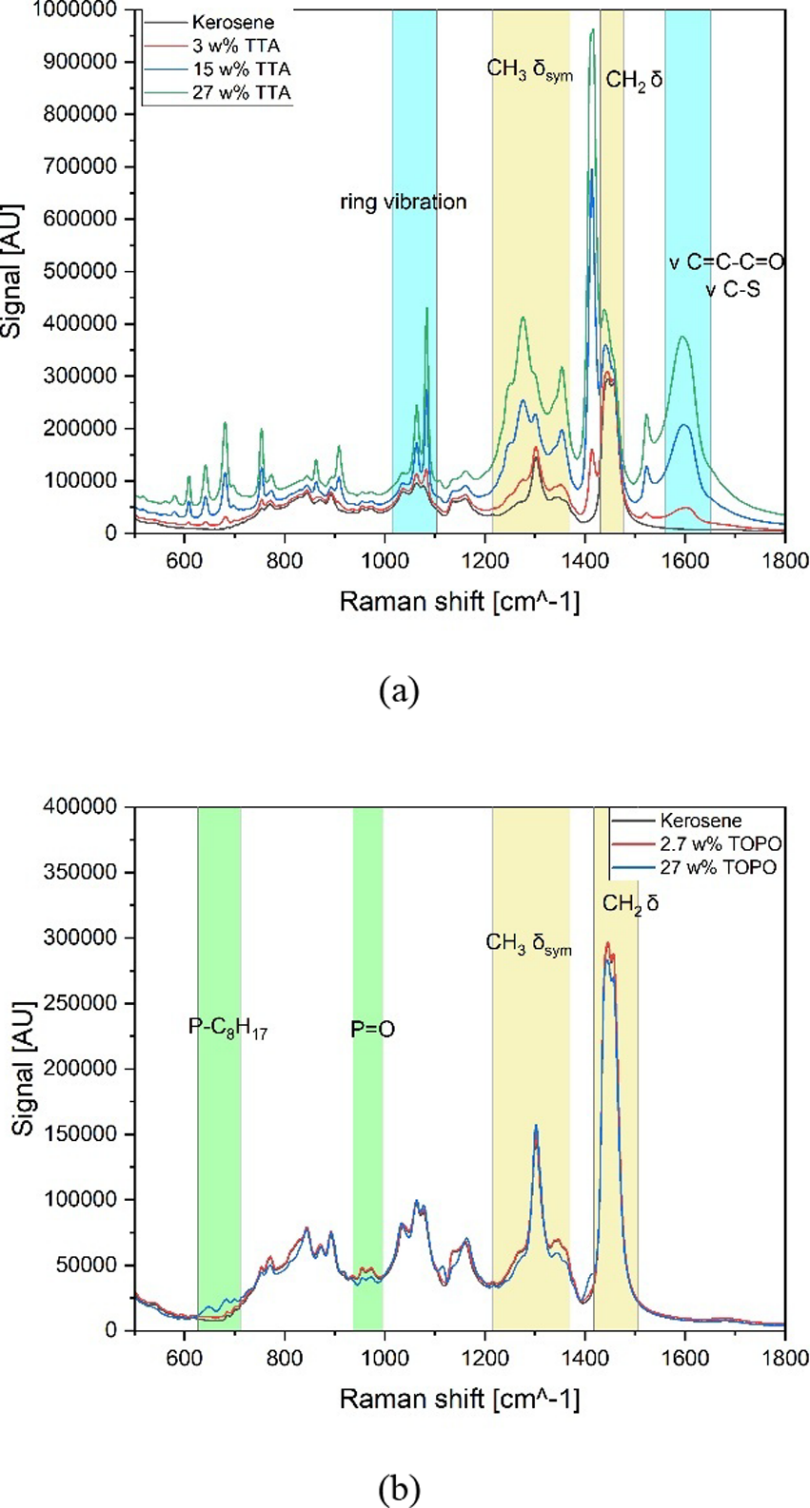
Raw Raman spectra of (a) TTA in kerosene and (b) TOPO in kerosene.

In both Raman spectra, the kerosene peaks show high intensities, especially within the TOPO sample set. For TTA, the ring vibration (around 1000 cm^–1^) and the C–S bands of thiophene (around 1600 cm^–1^) are highlighted since those bands were used as the major factor for the identification of TTA in different solutions due to their great recognition in other spectra as they show unique features of the TTA structure. The P–(C_8_H_17_) bonds (around 650 cm^–1^) and the P–O bond (in the region of 1000 cm^–1^) of TOPO are identified as the most important for the identification of this compound in kerosene.^64,66,67^ Attention has to be paid to the fact that the bands of TTA and kerosene are close to each other, and therefore, an overlap of bands could occur at higher TTA concentrations (greater than 20 w% TTA), especially for the Raman signal of the thienyl group, placed at 1414 cm^–1^, which is positioned between the two kerosene signals. Due to the potential overlapping of the TTA and kerosene bands, which might lead to difficulties of differentiation of these two compounds, the band at 1414 cm^–1^ is not further taken into consideration.

For the quantification of TTA and TOPO, the organic phase samples are combined as one sample set. The spectra are reduced to the region between 1780 and 780 cm^–1^, including the most important bands for TTA and TOPO. The data are preprocessed using SNV (for FT-IR analysis) and Savitzky–Golay derivative (first order) together with SNV for the Raman analysis. Figure 6 shows the reference concentrations of TTA and TOPO plotted against the predicted concentration by the PLS model using the FT-IR spectra. The regression is characterized by an *R* ^2^ of 0.97 and an RMSE of 12.8 g/L for TTA and a *R* ^2^ of 0.99 TOPO and an RMSE of 6.1 g/L for the validation set. The quality of the regression is reflected by the test set with an RMSE of 5.1 g/L for TTA and 8.2 g/L for TOPO. With similar *R* ^2^ and RMSEs in the validation and external test sets, an error in the regression training or validation can be ruled out.

**Figure 6:**
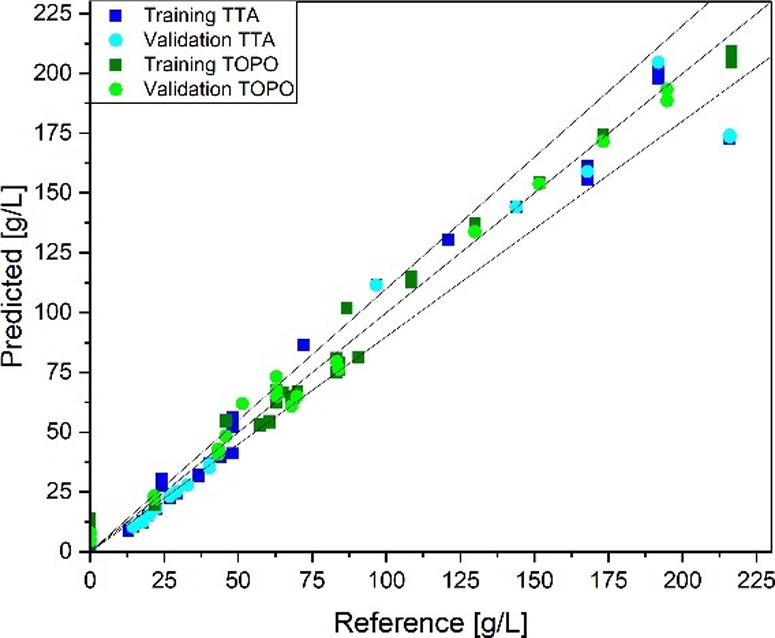
PLS regression parity diagram for the determination of TTA and TOPO concentrations from FT-IR spectra.

The PLS regression model based on the Raman spectra (see Figure 7) is similar to the FT-IR model. In the higher concentration range (concentrations bigger than 175 g/L), the Raman model shows poorer results compared to the FT-IR results. With an *R* ^2^ of 0.97 and an RMSE of 11.9 g/L for TTA and an *R* ^2^ of 0.99 and an RMSE of 3.7 g/L for TOPO, the Raman PLS regression model is comparable for the TTA quantification to the FT-IR based model. The test set yielded an RMSE of 6.0 g/L for TTA and 2.9 g/L for TOPO.

**Figure 7:**
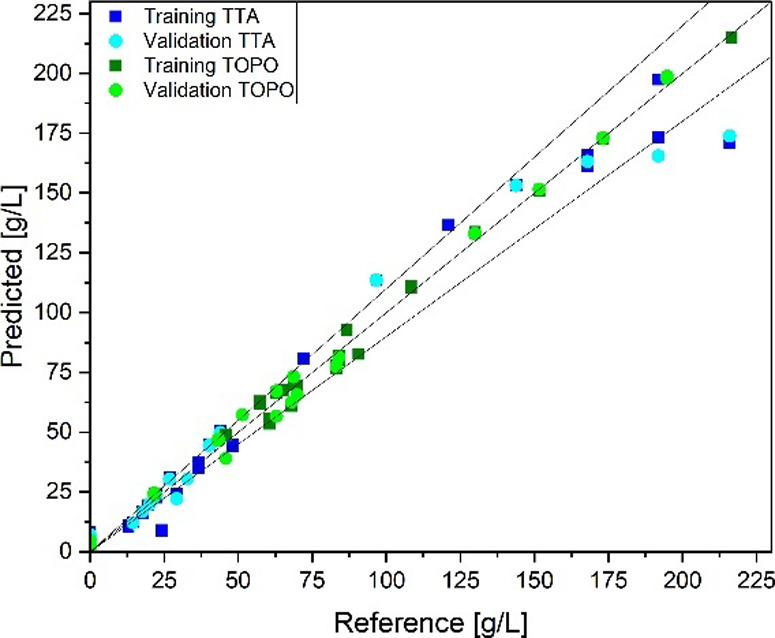
PLS regression parity diagram for the determination of TTA and TOPO concentrations from Raman spectra.

It can be stated that both measurement techniques are applicable for the quantification of TTA and TOPO in the organic kerosene phase and can be used for monitoring the composition of the organic phase.

### 4.2 Degree of Saponification

The samples for the determination of the degree of saponification are split into three different groups. Each group consisted of only one saponifier, which changes in concentration, and the degree of saponification ranges between 0 and 1. The organic phase, consisting of a constant TTA/TOPO ratio and concentration, is directly saponified. This is achieved by directly introducing NaOH or LiOH into the organic phase. To transfer the hydroxide ions produced by the partial dissociation of ammonia in water, the two phases are brought into contact for a sufficiently long time for mass transfer to be concluded. The phase separation is performed by using a centrifuge.

The organic phases are measured with FT-IR [for the spectra, see Figure 8a] and Raman [spectra shown in Figure 8b]. The wavenumber range of interest for FT-IR is between 1800 and 900 cm^–1^, while the Raman spectra range between 1000 and 1700 cm^–1^. In both figures, two spectra per saponifier are shown, representing the concentration ranges.

**Figure 8:**
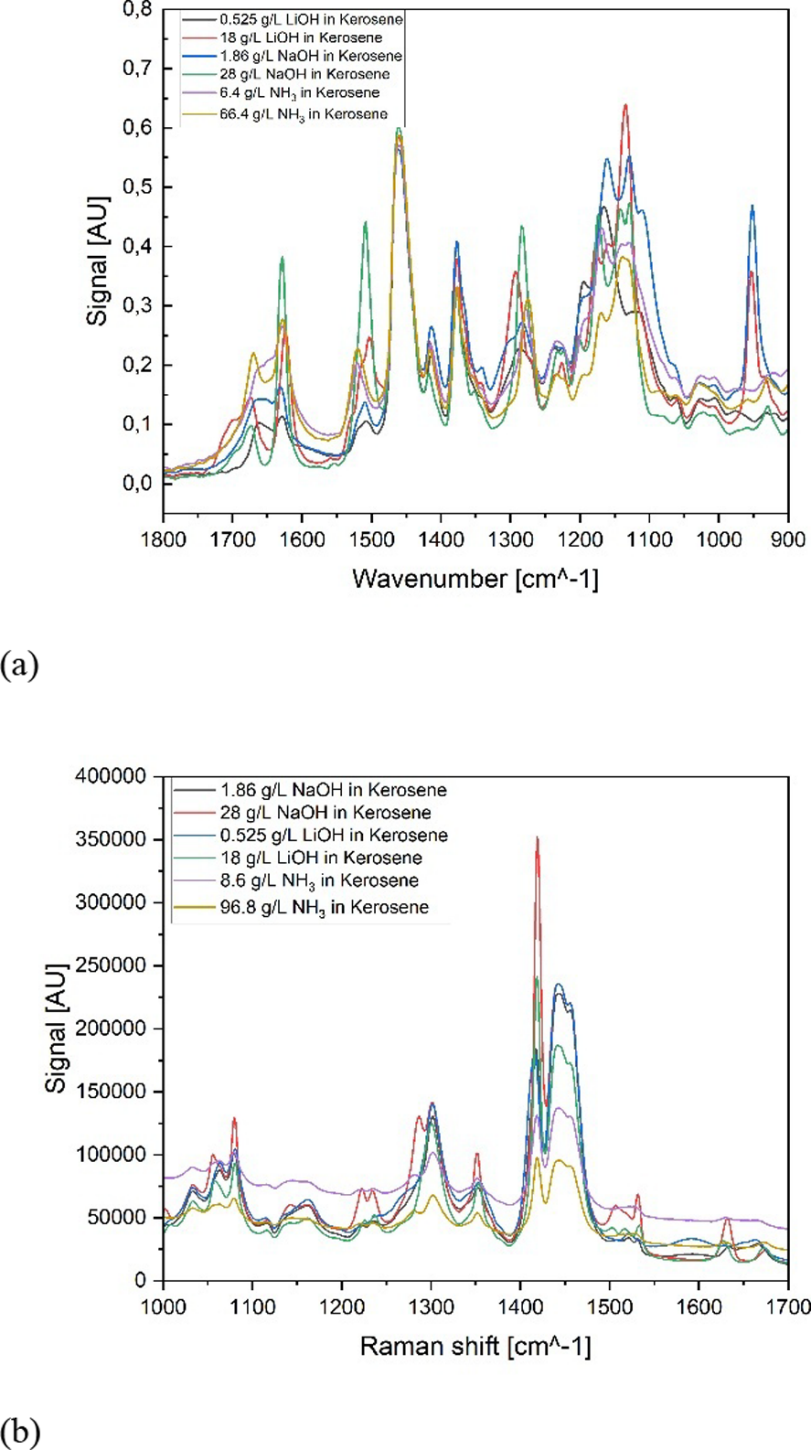
Raw spectra of saponified organic phase with NaOH, LiOH, and NH_3_ from (a) FT-IR and (b) Raman spectroscopies.

Depending on the chosen base, the spectrum shows significant differences, for example, in the region of 1300 cm^–1^. NaOH samples result in a band that is shifted to lower energies compared to the bands of LiOH, while the bands of ammonia are positioned between the other two bases. This might be caused by the different complexes formed during saponification and their slightly different energy distribution. Additionally, an increase in the signal intensity in connection with an increase in the base concentration can be observed [see Figure 8b at 1400 cm^–1^].

To reduce the influence of a single saponifier species or the cation of the saponifier and the possible formation of Me­[TTA­(TOPO)_2_] complexes, the resulting model is trained with the combined data set. The treatment of the bases on their own led to better results in comparison with the complete data set (see [Table tbl1]). In the FT-IR analysis, two bands, which show high significance in the calculation of the PLS regression, are placed at 1500 and 1300 cm^–1^. These two bands are expected to be only significant for the deprotonation of TTA. The band at around 1500 cm^–1^ (next to the kerosene band) is characterized by the deformation vibration of the C–OH and C–O groups. A change in this band indicates the deprotonation of TTA since the vibration of the C–O is more dominate in the structure than that of the C–OH group. This can be explained by the fact that the proton of the hydroxyl group is integrated into the intramolecular hydrogen bond and therefore not completely free in its vibration. The second band placed at 1300 cm^–1^ is mostly influenced by the ring vibration and the substituents at the thiophene. A change in the charge of the molecule leads to a change of charge distribution inside the molecule and therefore influences the vibrations. On the other side, the most outstanding band in Raman is placed around 1416 cm^–1^.

In Figure 9, the PLS result regression of the combined saponification experiments plotted against the reference data for the FT-IR analysis is given. An *R* ^2^ of 0.95 was reached with an RMSE of 0.09 in the validation with a test set RMSE of 0.08. The key figures for the regression are shown in [Table tbl1].

**Table 1:** Summary of All PLS Regressions from FT-IR and Raman Data

	FT-IR				Raman			
	*R* ^2^		RMSE		*R* ^2^		RMSE	
name	training	validation	training	validation	training	validation	training	validation
TTA in kerosene	0.98	0.97	8.6 g/L	12.8 g/L	0.97	0.97	9.5 g/L	11.9 g/L
TOPO in kerosene	0.99	0.99	6.6 g/L	6.1 g/L	0.99	0.99	3.3 g/L	3.7 g/L
degree of saponification	0.94	0.95	0.09	0.09	0.97	0.97	0.06	0.07
total metal-ion concentration (20–100 mmol/L)	0.96	0.96	4.9 mmol/L	5.0 mmol/L				
total metal-ion concentration (150–240 mmol/L)	0.90	0.93	4.2 mmol/L	2.9 mmol/L				
total metal-ion concentration					0.93	0.89	24.0 mmol/L	30.3 mmol/L

**Figure 9:**
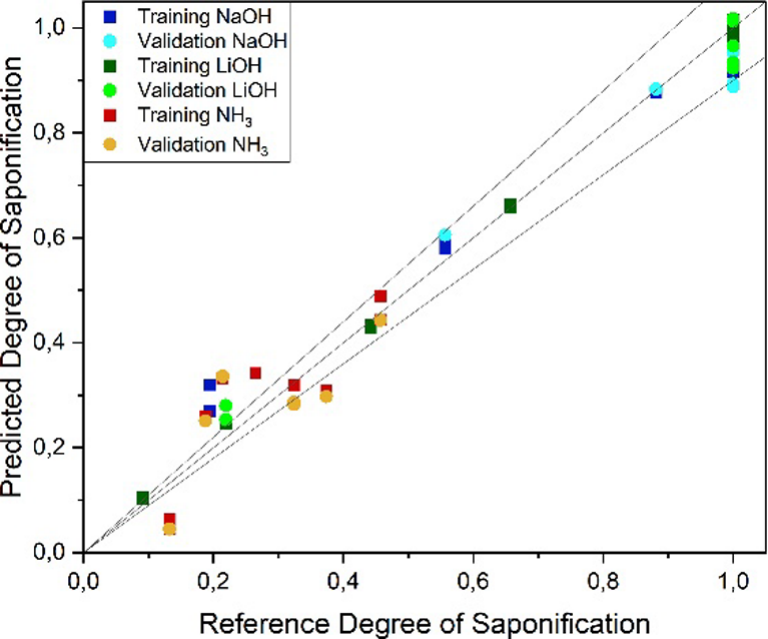
PLS regression parity diagram for the determination of the degree of saponification from FT-IR spectra.

For the Raman analysis, the resulting diagram is exemplified in Figure 10. In general, the Raman model reaches higher *R* ^2^ values of 0.97 and lower RSME values of 0.07 in the validation. Therefore, it can be concluded that Raman measurements are more suitable for the determination of the degree of saponification.

**Figure 10:**
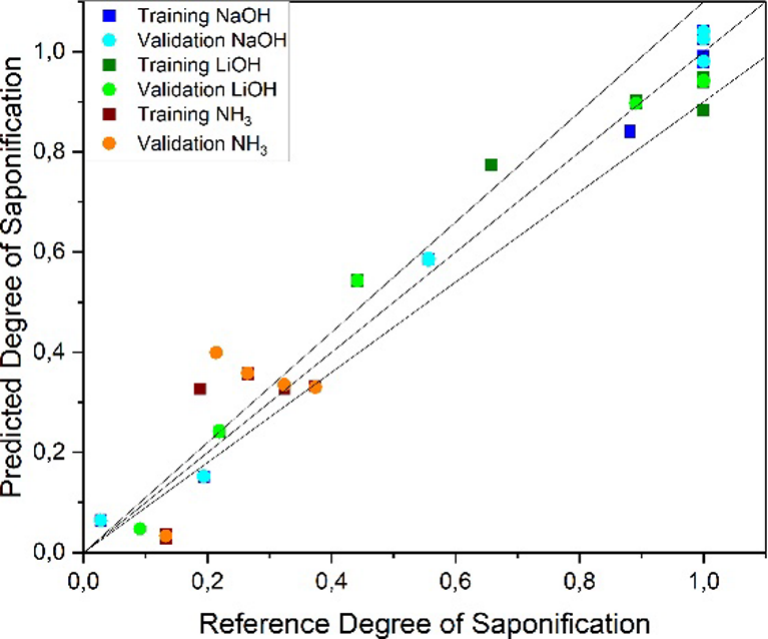
PLS regression parity diagram for the determination of the degree of saponification from Raman spectra.

### 4.3 Metal-Ion Complexes

The stripping of the loaded organic phases is performed by using H_2_SO_4_ at two different pH values. The resulting aqueous phases are analyzed with flame-AAS to identify the remaining metal-ion concentration in the organic phase. Figure 11 presents the FT-IR spectra of the organic phases after the stripping procedure. It can be seen that the spectra are mainly influenced by the remaining metal-ion concentration. The shape of the band at 1300 cm^–1^ is a good example of the spectral influence of the metal-ion complexes. With the low metal-ion complex concentration (or even no metal ions at all), the band shows a low intensity and a large peak width (around 60 cm^–1^), while the other shows a greater maximum height and a smaller peak width. Especially in the region of 1200 to 1100 cm^–1^, a significant band shift is indicated, which is linked to the metal-ion complex concentration. If the metal-ion complex concentration rises above 150 mmol/L, a band positioned at 1130 cm^–1^ can be identified in the spectra, while concentrations below show a significant band at 1160 cm^–1^. Possible differences linked to the use of NaOH or LiOH cannot be seen in the spectra.

**Figure 11:**
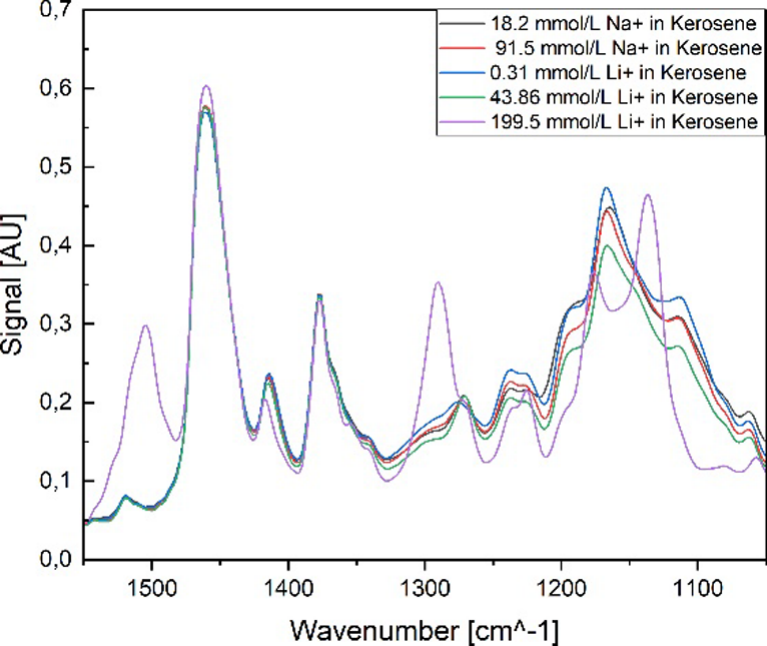
Raw FT-IR spectra of the organic phase with Na and Li complexes.

During the stripping of the organic phase, the metal ions are transferred from the organic phase to the aqueous phase. During this process, the Me­[TTA­(TOPO)_2_] complex, which is formed during the extraction from the original aqueous phase, will be decomposed. This decomposition can be defined by a shift in the bond type and bond length of P with O. In the Me­[TTA­(TOPO)_2_] complex, a P–O single bond is assumed. Zhang et al. describe the phenomenon of an increase in the bond length of P=O, leading to a partial formation of P–O bonds when the complexation took place. This indicates the participation of phosphorus and therefore TOPO in the formation of the Me­[TTA­(TOPO)_2_] complex.^50^ The occurring shift is also proven by Mansik et al. when dealing with phosphonic acids.^68^ They stated that the characteristic oscillation of the P=O bond at 1169 cm^–1^ (in this work, the band is identified at 1167 cm^–1^) decreases with an increasing organic metal concentration. On further increasing the metal concentration, they noticed the appearance of a second band at a lower wavenumber (here: at 1136 cm^–1^). Manski et al. were not able to correlate the band intensities with the free ion exchanger (containing the phosphonic acid) concentration. The band shift is described for different systems, for example, in a zinc/D2EHPA system.^69^ In Figure 12, the wavenumber region between 1220 and 1080 cm^–1^ is presented, in which the described phenomenon of the band shift occurs. The band positioned at 1167 cm^–1^ is assigned to the P=O vibration, while the P–O vibration is visible at 1136 cm^–1^.^9,64,68^

**Figure 12:**
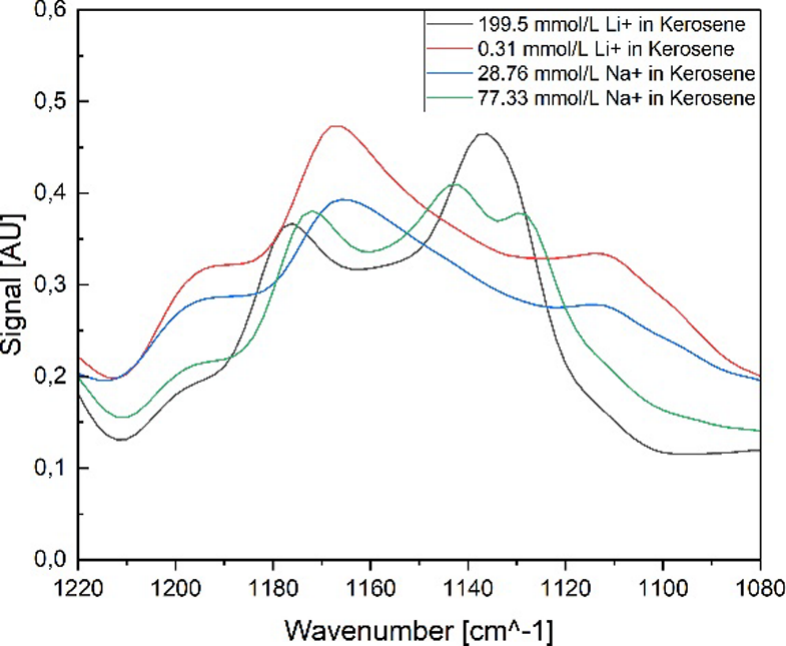
Raw FT-IR spectra in the peak shift region of the organic phase with Na and Li complexes.

A connection between the remaining metal ion concentration of the organic phases after stripping and the appearance of a P=O or P–O vibration band can be observed. As shown in Figure 2, this occurs in the TTA-TOPO-Me complex formation. The shift between the two bands for the lithium samples is well-defined. When the Li^+^ concentration is above 150 mmol/L, only the band at 1136 cm^–1^ is detectable; otherwise, the band at 1167 cm^–1^ is present inside the FT-IR spectra. For the Na^+^ concentration, a concentration range between 30 and 100 mmol/L is identified as an intermediate range. Some samples in this range show a band at 1167 cm^–1^, while others already display the band at 1136 cm^–1^.

Based on this shift and the applications of the resulting models, two concentration ranges are defined to be able to quantify the metal-ion concentration in the organic phases. The first sample group contained samples with concentrations between 20 and 100 mmol/L. The model will be used for stripped organic phases to support the process control. Inside Figure 13, the FT-IR PLS regression model based on low concentrations is presented. The model reached a quality of *R* ^2^ of 0.96 and RMSE of 5.0 mmol/L in the validation and RMSE of 8.1 mmol/L with the test set. In this model, no differentiation between Na^+^ or Li^+^ could be obtained.

**Figure 13:**
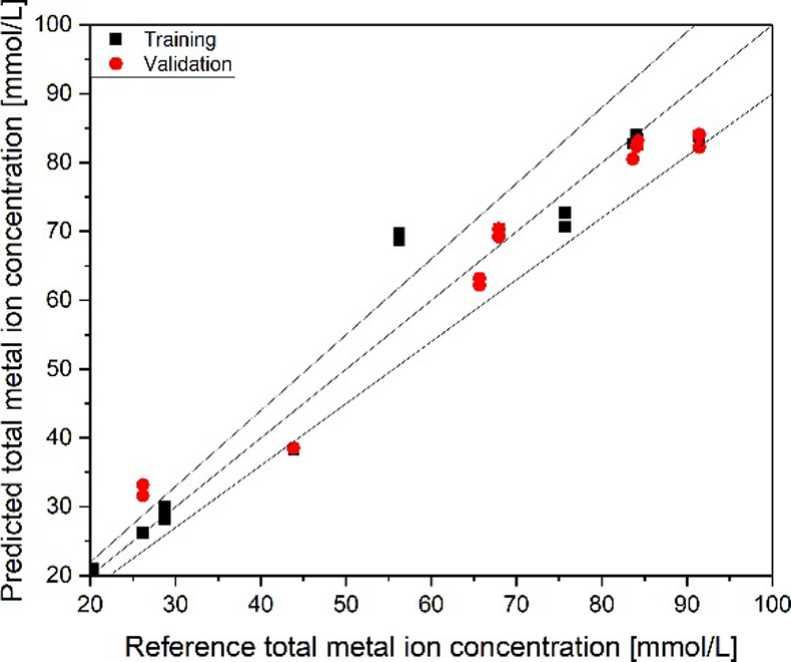
PLS regression parity diagram for the determination of metal-ion complex concentration from FT-IR spectra for the low-concentration region.

The results of the PLS regression model for higher concentrations (metal-ion content between 150 and 240 mmol/L) are presented in Figure 14. The model reached an *R* ^2^ of 0.93 and an RMSE of 2.9 mmol/L in the validation and an RMSE of 8.2 mmol/L with the test set. With this model, differentiation between Na^+^ and Li^+^ in the organic phase is successfully performed.

**Figure 14:**
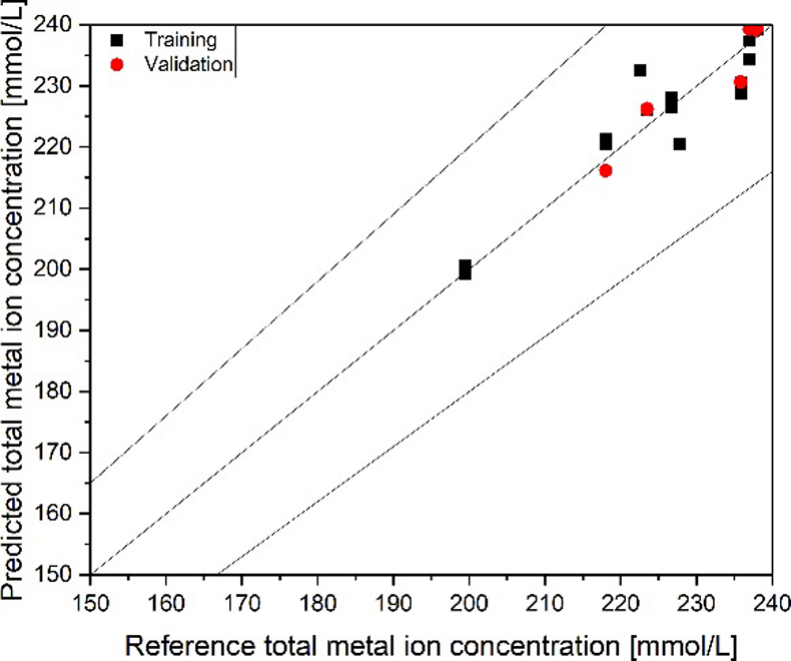
PLS regression parity diagram for the determination of metal-ion complex concentration from FT-IR spectra for the high-concentration region.

During the comparison of the organic phases at several preparation stages, two groups of bands are visible inside the spectra of the loaded organic phases (spectra are given in Figure 15). The first group appears in the range of 1200 to 1250 cm^–1^, while the second band group is located around 1500 cm^–1^. These bands are not visible in the pure organic phases as well as in the stripped phases. In the loaded organic phases, during the saponification and when extracting with pure water, the band groups can be identified inside the spectra. Due to this appearance, it is stated that the bands are related to the formation of the Me­[(TTA)­(TOPO)_2_] complex. Ahmed et al. analyzed the structures and spectroscopic information obtained by a new erbium complex formed with β-diketonate.^70^ Comparing the Raman spectra (shown in Figure 15) of the complex with the Raman spectra recorded during this study, some similarities can be identified. Especially the appearance of a double band in the region of 1200 to 1300 cm^–1^ is significant. Due to this parallelism, despite the fact of different metal ions and small structural differences in the second ligand, it can be stated that the bands are caused by the complex itself.

**Figure 15:**
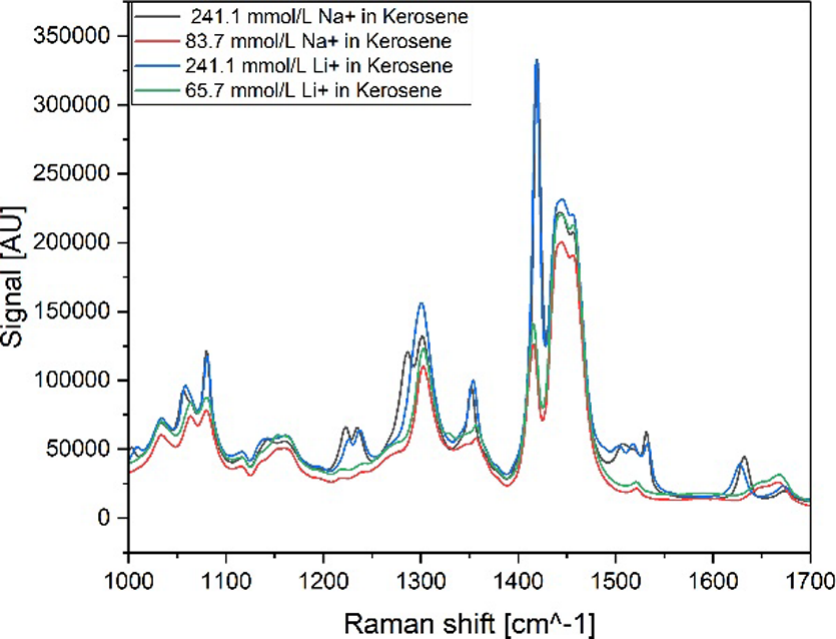
Raw Raman spectra of the organic phase with Na and Li complexes.

A differentiation of Na^+^ and Li^+^ inside the organic phase cannot be performed based on these two band groups.

Based on these spectra, the quantification of the metal-ion concentration is performed using PLS. The results of the PLS regression model for the total metal concentration are derived in Figure 16. The model shows some weaknesses in the concentration range of 20 to 100 mmol/L. When separating the data into the same concentration ranges, as demonstrated with the FT-IR data, the resulting PLS models are poorer by a factor of 5 compared to the model documented below. For the shown model, an *R* ^2^ of 0.89 and an RMSE of 30.3 mmol/L for the validation can be calculated, confirmed by an RMSE of 17.8 mmol/L in the test set. Therefore, the FT-IR model is more suited for the quantification of metal-ion complexes in the organic phase.

**Figure 16:**
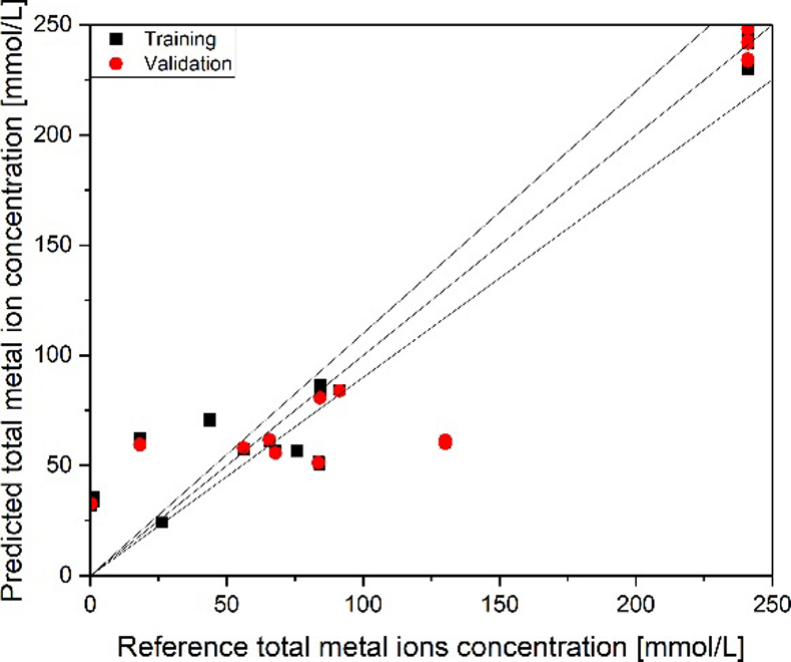
PLS regression parity diagram for the determination of the metal ion complex concentration from Raman spectra.

Table 1 summarizes the regression coefficients and RMSE achieved with FT-IR and Raman spectra through PLS regression. All sample spectra have also been resolved with the use of MCR-ALS, Table 2 summarizes for regression with MCR-ALS. These have in contrast to the PLS regression not been preprocessed but the same range of the spectrum was used. The resulting regression quality from the analysis to the measured chemical concentration can be found in Table 2 and in the Supporting Information section. Comparable results to the PLS regression could be achieved.

**Table 2:** Summary of All MCR-ALS Analyses from FT-IR and Raman Data

name	FT-IR		Raman	
	*R* ^2^	RMSE	*R* ^2^	RMSE
TTA in kerosene	0.98	8.53g/L	0.99	4.59 g/L
TOPO in kerosene	0.96	11.9 g/L	0.98	7.61 g/L
degree of saponification	0.87	0.134	0.97	0.03
total metal-ion concentration (0–100 mmol/L)	0.93	8.76 mmol/L		
total metal-ion concentration (150–240 mmol/L)	0.77	6.09 mmol/L		
total metal-ion concentration			0.92	27.7 mmol/L

Harvianto et al. published results on the extraction of lithium using the TTA/TOPO system. It is observed that the extraction efficiency decreases with the recycled organic phase.^39^ For other β-diketones, the loss from the organic phase during extraction using total organic carbon (TOC) analysis has already been demonstrated.^7,71^ In this work, aqueous phases are prepared at acidic, basic, and neutral pH values, and attempts are made to dissolve TTA or TOPO in them. FT-IR spectra in Figure 17 show that in all cases, no bands characteristic of TOPO are visible. Several peaks in the range of 1300 to 1050 cm^–1^ are visible in the acidic solutions, which can be attributed to the SO_4_^2^^–^ ions. Only in the basic solution are peaks visible in the TTA sample, which can be clearly attributed to the characteristic bands of TTA. It can therefore be confirmed that TTA dissolves in water in a basic environment. This occurs during the extraction of lithium, which is why the extraction efficiency decreases with a recycled extraction agent.

**Figure 17:**
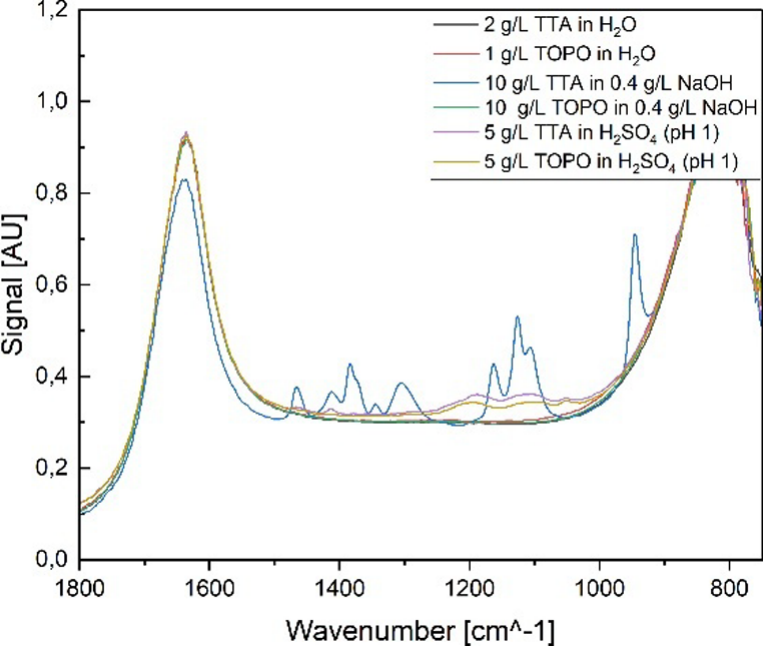
Raw FT-IR analysis of aqueous phases with various simulated pH values and either TTA or TOPO.

## 5 Discussion

From a comparison of the spectroscopic techniques, it can be seen that the *R* ^2^ and RMSE of the PLS are comparable from FT-IR and Raman spectra when determining both TTA and TOPO concentrations. For the degree of saponification, the Raman analysis delivers a better validation and RMSE than FT-IR. When determining the metal complex concentration, a better regression result can be achieved with FT-IR and separate concentration ranges than with Raman. Notably Raman outperformed FT-IR when analyzed with MCR-ALS.

The regression coefficients and RMSE for TTA and TOPO quantification are comparable from PLS to MCR-ALS for FT-IR and Raman. For the metal-ion complex and the degree of saponification quantification from FT-IR spectra, a difference between PLS and MCR-ALS can be detected. Both MCR-ALS and PLS are regularly used in reactive liquid–liquid -extraction applications.^72–76^ In other applications, similar results between PLS and MCR-ALS have been achieved.^77,78^

Thus, either FT-IR or Raman can be used to determine the concentrations of TTA and TOPO, and Raman can be used for the degree of saponification. Criteria such as acquisition costs and operating and maintenance costs are important for the decision. For the proposed integration of the PAC systems, FT-IR and Raman systems are chosen here. This is developed in Figure 18. PAC in-line spectroscopy can be used in four process operation steps:

1. When preparing the organic phase, the concentration of TTA and TOPO, as well as the ratio of the components, can be adjusted. The aim is to achieve the highest possible concentration of TTA and a ratio of TOPO to TTA of 2:1. Continuous adjustment of the organic phase can be particularly advantageous when recycling the organic phase, especially with the shown loss of TTA.
2. During saponification, NaOH is added to the organic phase. The aim is to achieve a saponification degree of approximately one. Adding NaOH beyond the saponification degree does not increase the efficiency and may make scrubbing or stripping less efficient, thus incurring high costs. Adding too little can make the process less efficient and lead to yield losses.
3. During extraction, the phase ratio can be adjusted via feed addition by analyzing the metal-ion complexes in the organic phase so that extraction efficiency is maximized. The model with good accuracy in the high-concentration regime is used.
4. During stripping, similar to extraction, the metal-ion complexes in the organic phase can be measured, and the phase ratio in stripping can be continuously adjusted. The goal here is to achieve as complete a transfer of the metal ions into the aqueous phase as possible. The model with good accuracy in the low-concentration regime is used.

**Figure 18:**
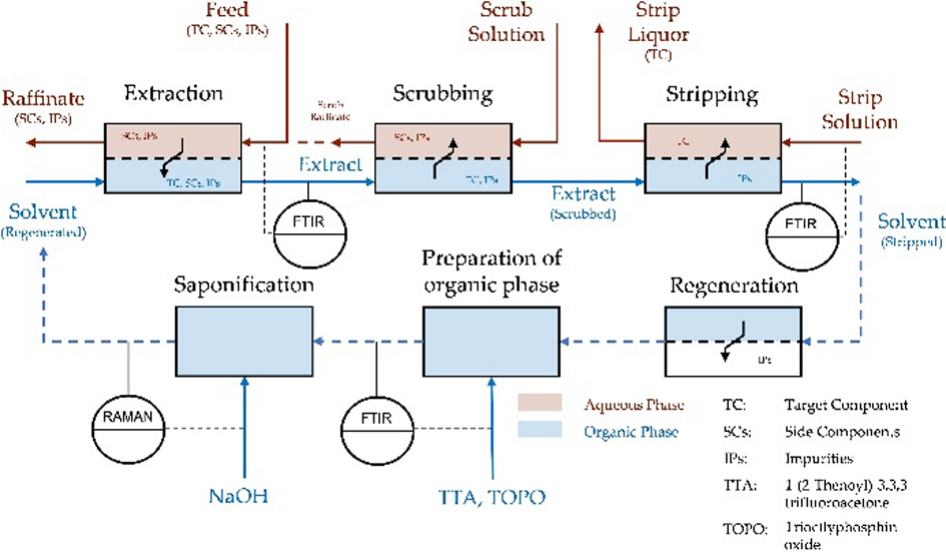
Proposed PAT integrated control circuit.

The proposed case of the determination of metal-ion complexes is two-fold. First in the extraction stage from the feed where as high as possible concentration is desired; a low-concentration regime is not applicable here. Second, in the stripping step, a low concentration is desired, so a model suitable for the high-concentration regime is not applicable here.

Most of the operating costs associated with solvent extraction arise from the high consumption of alkalis for saponification and acids for stripping. These costs exceed the costs of extraction agents, energy requirements, and labor costs.^79–81^ Excessive use of acid, base, and extraction agents impede the economic feasibility of recycling.^82,83^ A QbD-based process design relying on the described PAC systems as PAT to precisely control the dosages of these chemicals can have an economic and ecological advantage. As an example, the extraction of lithium from a brine of lithium and sodium is used to show potential economic and ecological gains:

When extracting lithium in a continuous process at a rate of 0.165 t/h (8000 h/a operating time) with a β-diketone, the annual cost of NaOH is estimated to $5.8 million.^7^ In the process presented, with the recovery of the extraction agent, this corresponds to 62% of the cost of the chemicals used or 22% of the total OPEX. These costs arise primarily in saponification.^84^ Similar results were obtained for the β-diketone dibenzoylmethane (HDBM), where 55% of the chemical costs consisted of NaOH and 18% of H_2_SO_4_. For the β-diketone HBTA, the costs of the extractant predominate due to the loss during extraction, which is therefore less economical. The consumption of NaOH and H_2_SO_4_ is also strongly reflected in the life cycle assessment, in which approximately 30% of the GWP is caused by acid and lye consumption.^7^ Precise regulation of the use of these chemicals is therefore an economic and ecological advantage.

The RMSE of the determination of the saponification degree with Raman-PLS is 0.07; therefore, the maximum yield in the extraction can be achieved with an excess supply of 7%. If an excess dosage of 30% is assumed for comparison, this represents a reduction in chemical costs of 15% for the saponification step. For stripping, an RMSE of the metal-ion concentration of 8.2 mmol/L is calculated in this work. If a similar argument is applied to acid consumption, then a further reduction in chemical costs of 8% can be demonstrated for the stripping step. For a plant with an annual production of about 1.3 kt/a Li, this amounts to savings of roughly $500,000 per year. With a purchase price of about $100,000 for an FT-IR^85^ and roughly a similar amount for Raman,^86,87^ a return on investment (ROI) of less than 0.4 years can be calculated for the monitoring and control of acid and lye dosing. This could save a total of about 20% in GWP based on the data of Jieun-Cha et al.^7^

A high concentration of extractants increases the efficiency and productivity of the separation process, but oversaturation leads to waste of the extraction agent,^51^ which is why accurate redosing is advantageous. Loss of extraction agent during the extraction stage is already known for the β-diketone.^7,71^ Acid re-extraction can be carried out to recover up to 89%, which greatly increases the economic efficiency of the process.^84^ The loss of extraction agent was measured using a total organic carbon measurement;^7,84^ quantification can also be performed using FT-IR, as shown here. The advantage here is that the concentration can be measured directly in the organic phase and does not have to be inferred from the concentration of the aqueous phase, which can be prone to errors.

Following the argument that control via pH value is subject to inherent uncertainty, whereas control via stoichiometric dosing is more efficient, conventional control via pH value can play a subordinate role or even be replaced altogether.^88^ This technology enables direct control of the hydrometallurgical process. In a process with the recycled organic phase and extraction agents, the concentration may have to be adjusted,^7,84^ so a PAT solution has its merits. The same argument is true for the saponification and formation of metal-ion complexes. Furthermore, the technology can be used for a more rapid process characterization in terms of kinetic parameter as demonstrated with the PUREX process.^58^ Integrating these PAT solutions into a process coupled with an initial off-line analysis campaign will also lead to a more precise PLS model as more data for retraining are available. For the application of the PAT system shown here into another purification step, another extractant requiring a new calibration is necessary.

Potential typical drawbacks of any in-line method are maintenance efforts like calibration, and actions to sensor drift and aging could be easily overcome by checking the baseline in each cycle of pure component phases of process operation and monitoring any drift in order to be able to react. Drift is prevented, and any sensor aging is detected a priori. Intervals of recalibration of the baseline have to be determined depending on the device integrated and tested during process development. Testing and validation of intervals can occur on a lab scale. Changes in the baseline can also be due to a change in temperature, which is easily identified and corrected for by shortening the interval accordingly. Another potential problem can arise from impurities from the feed in the form of previously not included ions or organic chemicals. These would have to reach a high enough concentration to have a significant effect on the spectra. This is possible with a continuously recovered organic phase. Although these are not included in the original calibration, PCA or MCR analysis can identify them as not previously known spectra. This will result in detection by the PAT system, which can then act accordingly. If set in relation to benefits, then a clear recommendation to follow the path to industrial implementation results. All proposed measurements can be taken as online instead of in-line measurements for periodic baseline checks and calibration.

## 6 Conclusions

In this work, FT-IR and Raman based PLS and MCR-ALS models have been calibrated and validated to measure in-line the concentration of extractants, the degree of saponification, and the concentration of metal-ion complexes in the organic phase as the critical product quality attributes of a Li extraction process. All PLS models achieve a sufficient *R* ^2^ and RMSE to quantify critical process parameters in all four processing steps: preparation of organic phase, saponification, extraction, and stripping of a continuous process in real time. With these PAT in-line spectroscopy systems, an integrated cycle is proposed and a GWP reduction by implementation up to 20% is calculated. The implementation can reduce the cost of chemicals of the process by 15%, which amounts to an ROI of less than 0.4 years.

Since control is based on phase ratios and the concentration of the extraction agent, it is independent of the equipment used for phase contraction and separation. Integration into model predictive control enabled by digital twins for mixer separators,^89^ centrifugal extractors,^90^ or columns^35^ is possible.

## Supplementary Material



## Notes

Conclusion table for all PLS regression models; conclusion table for MCR regression; additional information on the FT-IR analysis including preprocessed spectra, overview of the PCA analysis, and explained variances of the PLS regression (all of the following subtopics: TTA and TOPO analyses; degree of saponification; total metal concentration); additional information on the Raman analysis including preprocessed spectra, overview of the PCA analysis. and explained variances of the PLS regression (all of the following subtopics: TTA and TOPO analyses; degree of saponification, total metal concentration); quality of MCR-ALS regression diagrams for TTA and TOPO from FT-IR spectra and Raman spectra; degree of saponification from FT-IR and Raman spectra; and metal-ion complex concentration (PDF)

## Abbreviations

AAS: Atom Adsorption Spectroscopy
CRMA: Critical Raw Materials Act
FT-IR: Fourier Transform Infrared
GWP: Global Warming Potential
HBTA: benzoyl-1,1,1-trifluotoacetone
HDBM: dibenzoylmethane
HFTA: 4,4,4-trifluoro-1-furoyl-1,3-butanedione
ICP-MS: inductively coupled plasma mass spectrometry
ICP-OES: inductively coupled plasma optical emission spectroscopy
LIB: Lithium-Ion-Battery
LLE: Liquid–Liquid Extraction
MCR-ALS: multivariate curve resolution - alternating least-squares
MIR: Midinfrared
NIR: Near-Infrared
OPEX: Operational expenditure
PAC: Process analytical chemistry
PAT: Process Analytical Technologies
PCA: Principal Component Analysis
PUREX: Plutonium Uranium Reduction Extraction
PLS: Partial Least-Squares
QbD: Quality-by-design
RMSE: Root-Mean-Squared Error
ROI: Return of Investment
SNV: Standard Normal Variate
TOPO: trioctylphosphine oxide
TTA: 1-(2-thenoyl)-3,3,3-trifluoroacetone
XRD: X-ray diffraction
XRF: X-ray fluorescence
