# Supplementary material for: Process Analytical Technology-Integrated FT-IR and Raman Spectroscopies for Efficient Reactive Liquid–Liquid Extraction Processing in Lithium Recycling
Source: ACS Omega. 2025 Oct 20;10(42):49856–69. doi: 10.1021/acsomega.5c05705 (PMC12573149; doi:10.1021/acsomega.5c05705)
Supplement: Supplementary file 1 [file ao5c05705_si_001.pdf]

Supporting Information

Process Analytical Technology (PAT)-integrated FT-IR and Raman Spectroscopy for Efficient Reactive Liquid–Liquid Extraction Processing in Lithium Recycling

Alexander Uhl, Alexandra F. Humann, Axel Schmidt and Jochen Strube*

Institute for Separation and Process Technology, Clausthal University of Technology, Clausthal-Zellerfeld, Germany

Name	Number	Page
**Section 1:** **Conclusion Tables**		1-2
For all PLS regression models	S1, S2	1
For MCR regression	S3, S4	1-2
**Section 2:** **Additional information on the FT-IR analysis including preprocessed spectra, overview of the PCA analysis and explained variances of the PLS regression**		2-8
TTA TOPO analysis	S5 – S7	2-4
Degree of Saponification	S8 – S10	5-6
Total metal concentration	S11 – S14	6-8
**Section 3:** **Additional information on the Raman analysis including preprocessed spectra, overview of the PCA analysis and explained variances of the PLS regression**		9-13
TTA TOPO analysis	S15 – S17	9-10
Degree of Saponification	S18 – S21	10-12
Total metal concentration	S22 – S24	12-13
**Section 4:** **Quality of MCR-ALS regression diagrams**		14-16
TTA and TOPO from FT-IR spectra and Raman spectra	S25, S26	14
Degree of saponification from FT-IR and Raman spectra	S27	15
Metal ion complex concentration	S28 – S30	15-16

Section 1: Additional information of the PLS and MCR regression

Table S1: Conclusion of all PLS regressions from FT-IR data.

Name	**FT-IR**						
	R^2		RMSE		Pretreatment	Factors	Sample Size
	Training	Validation	Training	Validation			
TTA in Kerosene	0.98	0.97	8.6 g/L	12.8 g/L	SNV (range: 1780-780)	4	104
TOPO in Kerosene	0.99	0.99	6.6 g/L	6.1 g/L	SNV (range: 1780-780)	4	104
Degree of Saponification	0.94	0.95	0.09	0.09	SGolay (1st order), SNV (range: 1800-750)	5	76
Total metal ion concentration (20 – 100 mmol/L)	0.96	0.96	4.9 mmol/L	5.0 mmol/L	SGolay (1st order), SNV (range: 1220-1062)	5	36
Total metal ion concentration (150 – 240 mmol/L)	0.90	0.93	4.2 mmol/L	2.9 mmol/L	SGolay (1st order), SNV (range: 1220-1062)	4	27

Table S2: Conclusion of all PLS regressions from Raman data.

Name	**Raman**						
	R^2		RMSE		Pretreatment	Factors	Samples Size
	Training	Validation	Training	Validation			
TTA in Kerosene	0.97	0.97	9.5 g/L	11.9 g/L	SGolay (1st order), SNV (range: 700-1800)	5	104
TOPO in Kerosene	0.99	0.99	3.3 g/L	3.7 g/L	SGolay (1st order), SNV (range: 700-1800)	5	104
Degree of Saponification	0.97	0.97	0.06	0.07	SGolay (1st order), SNV (range: 1000-1700), outlier removed (3 samples with 6.4 g/L NH_3 in aqueous phase)	5	73
Total metal ion concentration	0.93	0.89	24.0 mmol/L	30.3 mmol/L	SGolay (1st order), SNV (range:1000-1700)	5	76

Table S3: Conclusion of all MCR regressions from FT-IR data.

Name	**FTIR**				
	R^2	RMSE	Wavenumber range	Components	Samples Size
TTA in Kerosene	0.98	8.53 g/L	700-1800 cm^{-1}	3	104
TOPO in Kerosene	0.96	11.9 g/L	700-1800 cm^{-1}	3	104
Degree of Saponification	0.87	0.134	1000-1700 cm^{-1}	3	73
Total metal ion concentration (0 – 100 mmol/L)	0.93	8.76 mmol/L	1220-1062 cm^{-1}	4	36
Total metal ion concentration (150 – 240 mmol/L)	0.77	6.09 mmol/L	1220-1062 cm^{-1}	4	27

Table S4: Conclusion of all MCR regressions from Raman data.

Name	**Raman**				
	R^2	RMSE	Raman shift region	Components	Samples Size
TTA in Kerosene	0.99	4.59 g/L	700-1800 cm^{-1}	3	104
TOPO in Kerosene	0.98	7.61 g/L	700-1800 cm^{-1}	3	104
Degree of Saponification	0.97	0.03	1000-1700 cm^{-1}	3	73
Total metal ion concentration	0.92	27.7 mmol/L	1000-1700 cm^{-1}	4	76

Section 2: Supporting information for FT-IR measurements

2.1 TTA and TOPO measurements

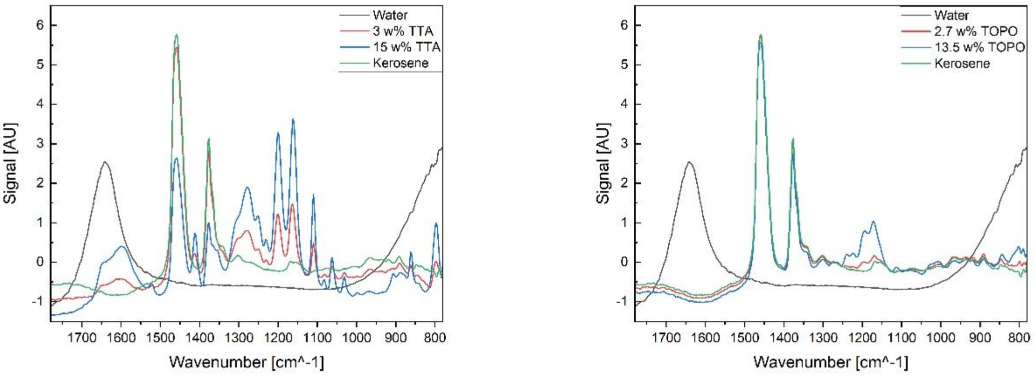

Figure S5: Preprocessed FT-IR Spectra of TTA in kerosene (left) and TOPO in kerosene (right).

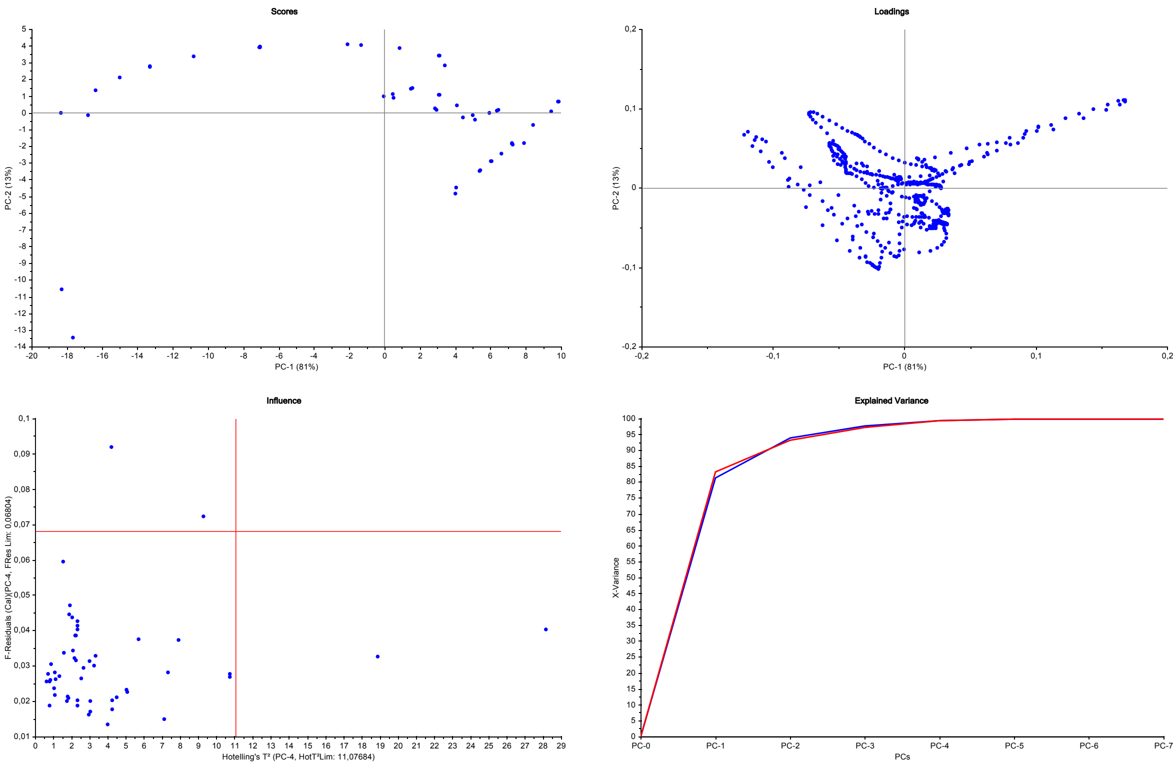

Figure S6: Summarized overview of the PCA after preprocessing.

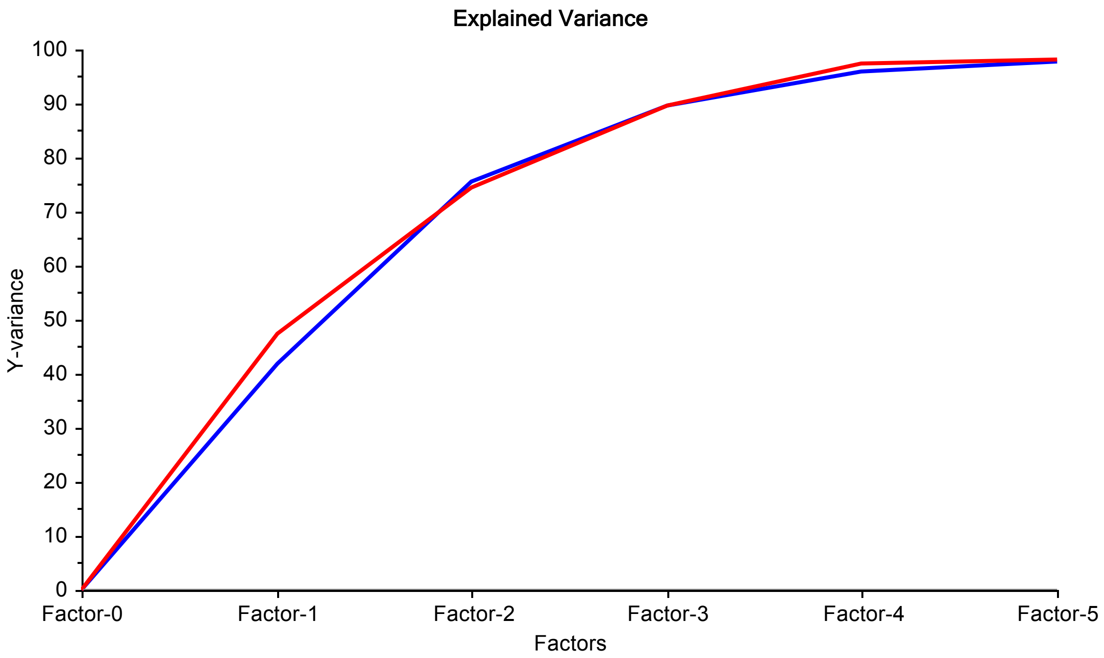

Figure S7: Explained variance by the regression factors of the PLS analysis.

2.2 Determination of Degree of Saponification

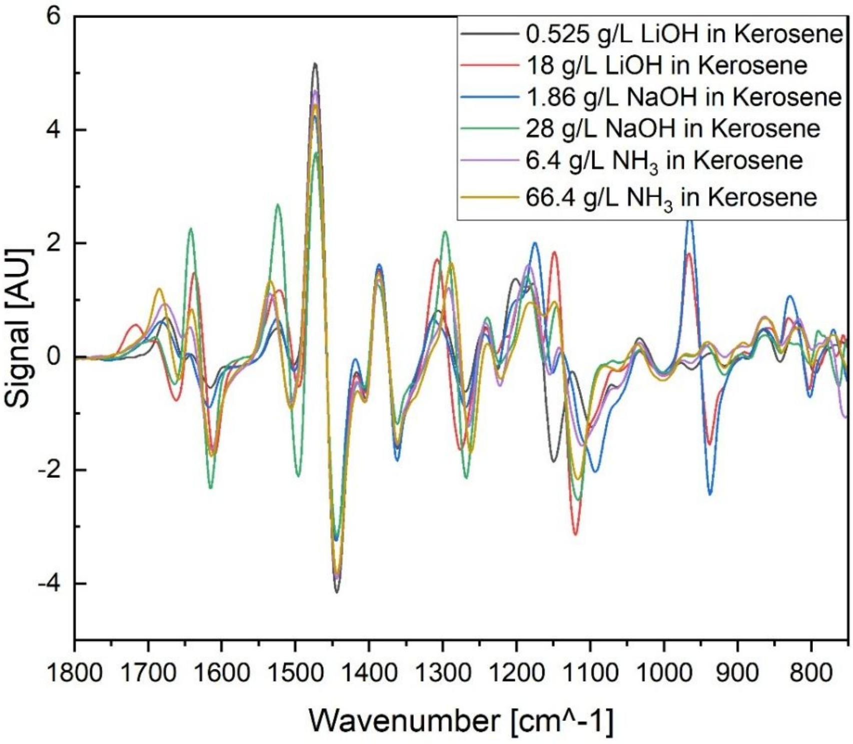

Figure S8: Preprocessed spectra of saponificated organic phase with NaOH, LiOH and NH_3 from FT-TR.

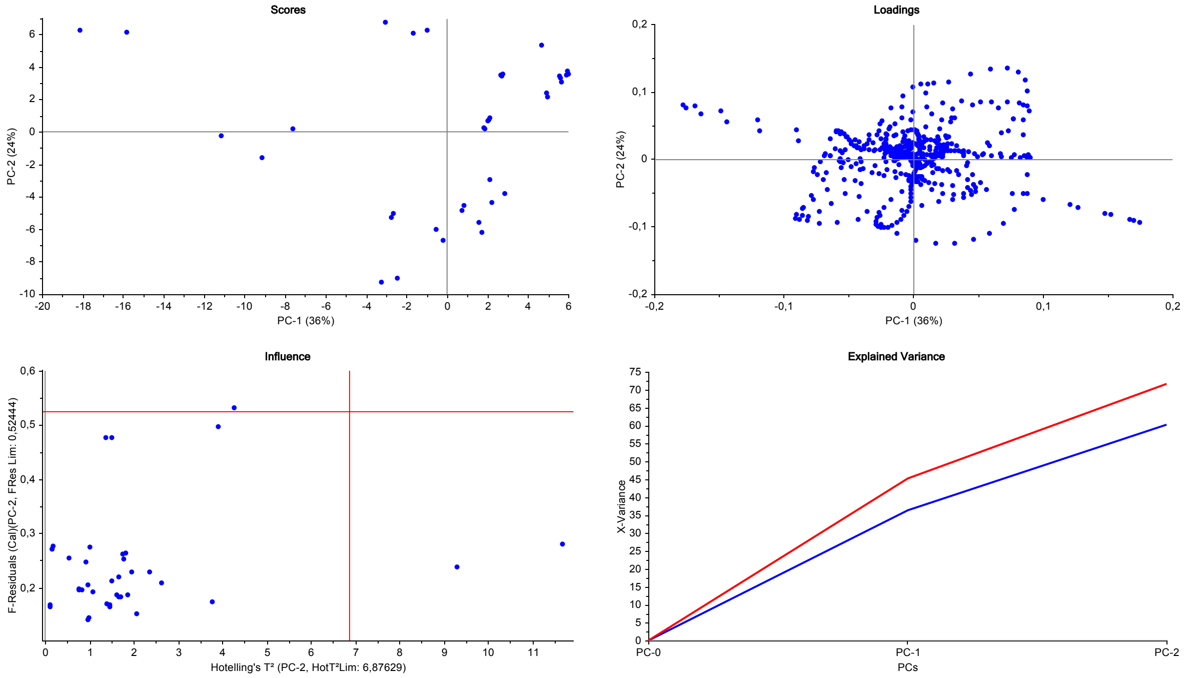

Figure S9: Summarized overview of the PCA after preprocessing.

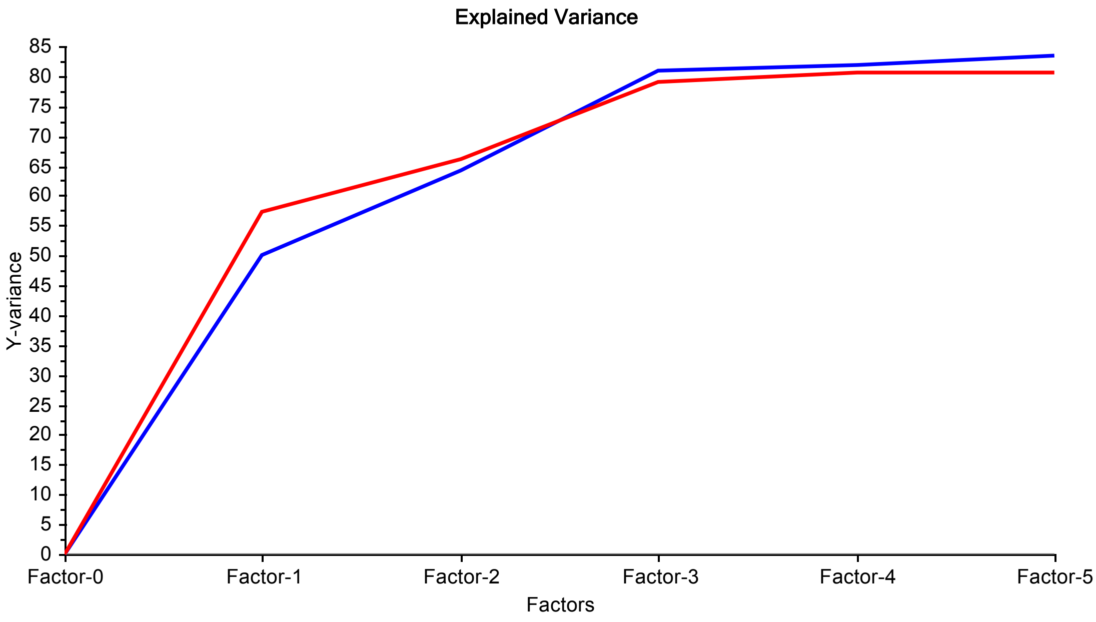

Figure S10: Explained variance by the regression factors of the PLS analysis.

2.3 Total metal ion concentration

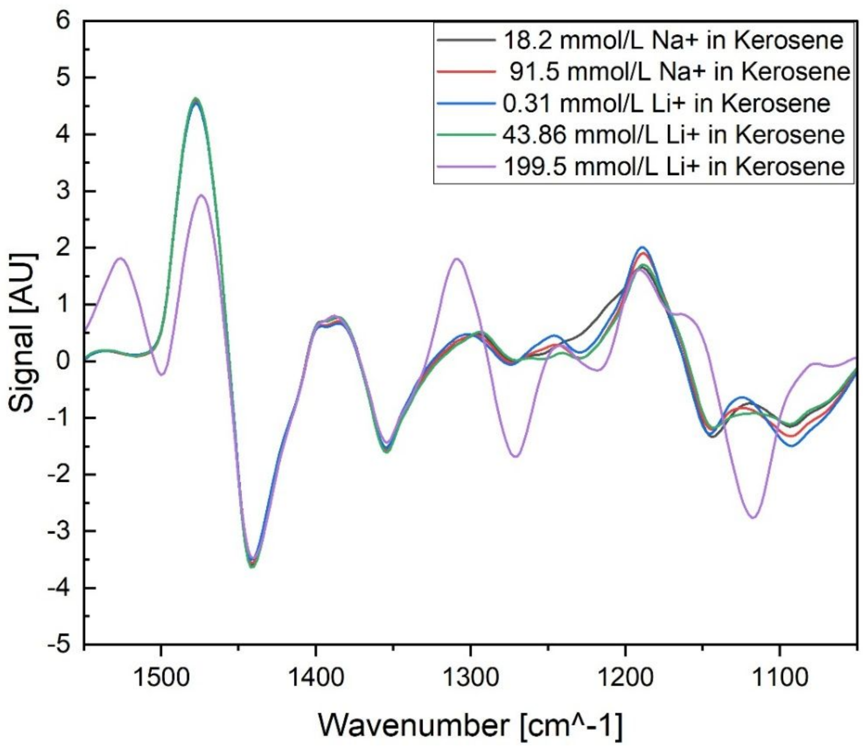

Figure S11: Preprocessed FT-IR spectra of organic phase with Na and Li complexes.

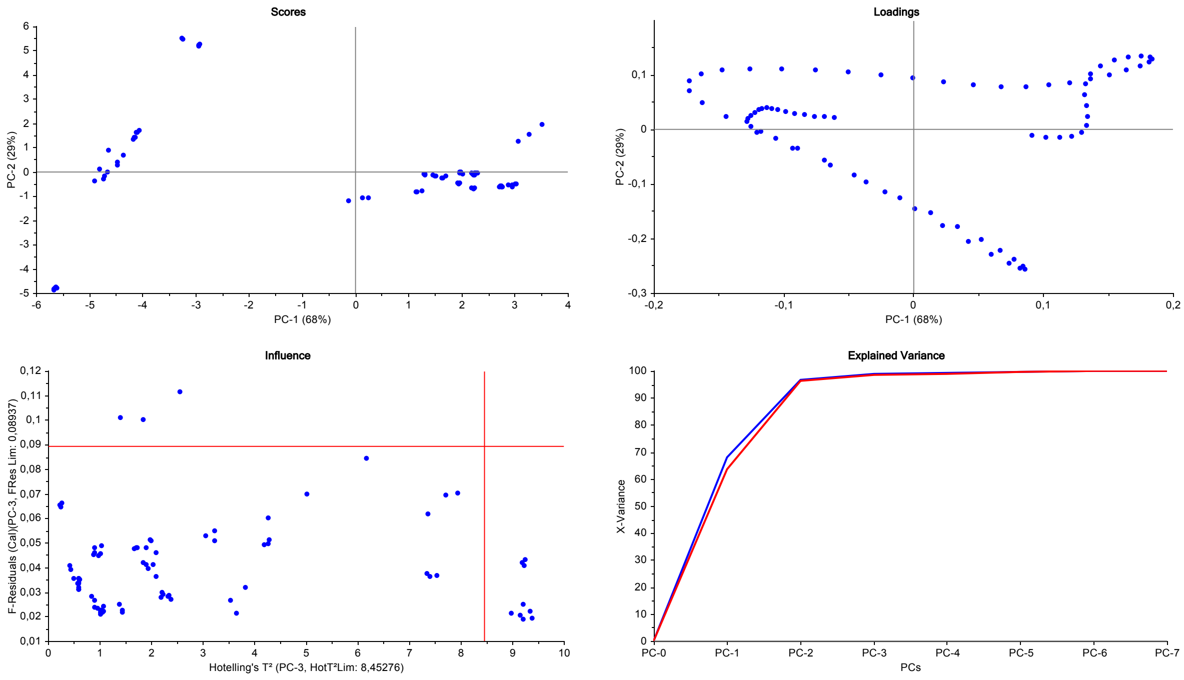

Figure S12: Summarized overview of the PCA after preprocessing.

2.3.1 Concentration range 20 – 100 mmol/L

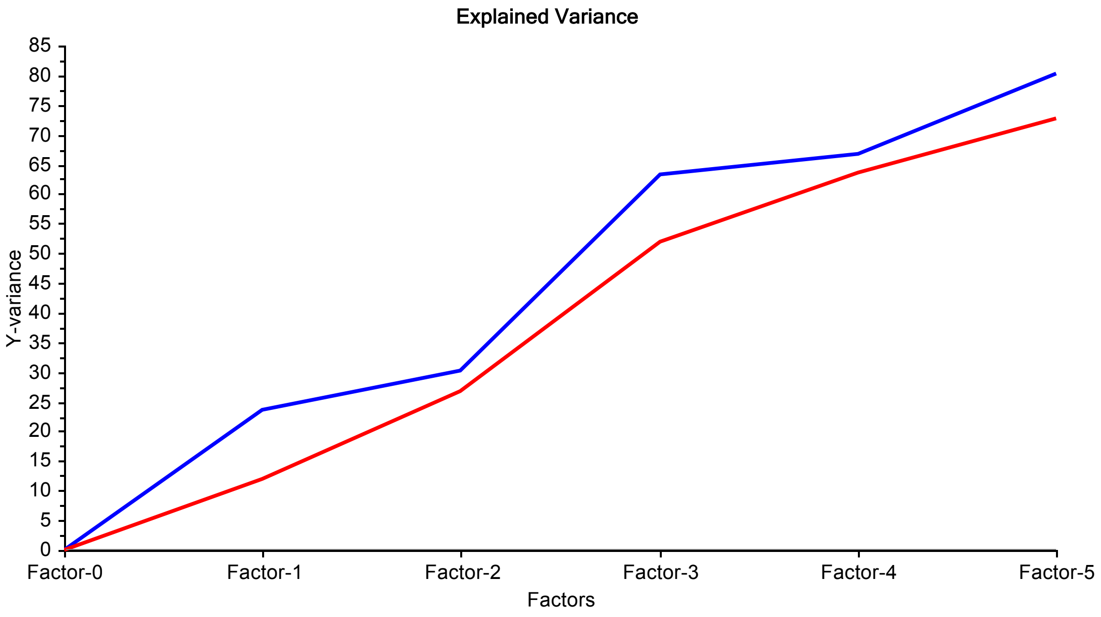

Figure S13: Explained variance by the regression factors of the PLS analysis.

2.3.2 Concentration range 150 – 240 mmol/L

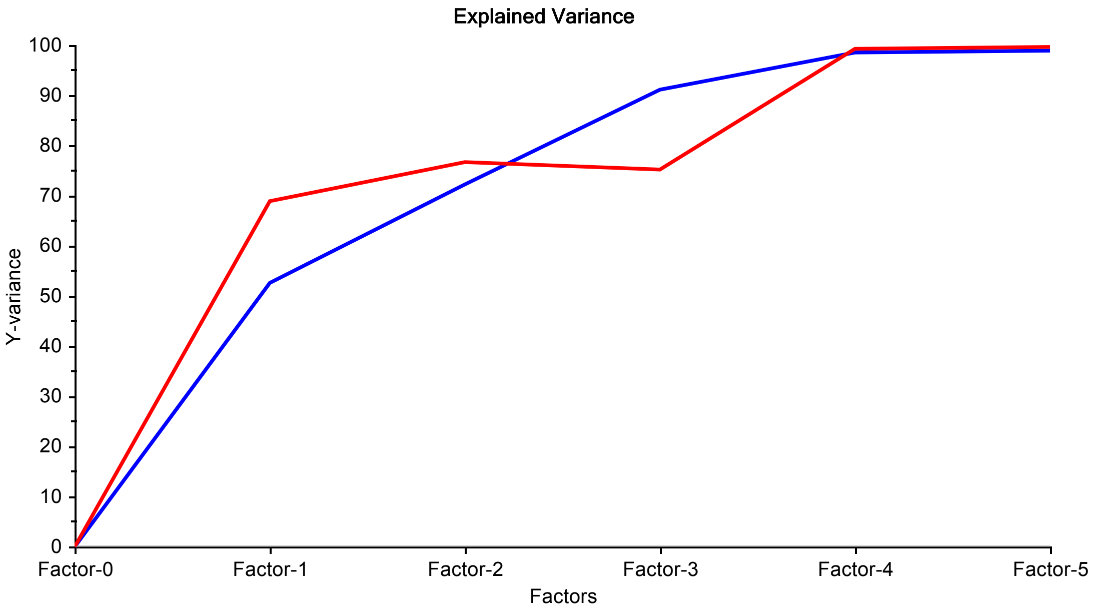

Figure S14: Explained variance by the regression factors of the PLS analysis.

Section 3: Supporting information for Raman measurements

3.1 TTA and TOPO measurements

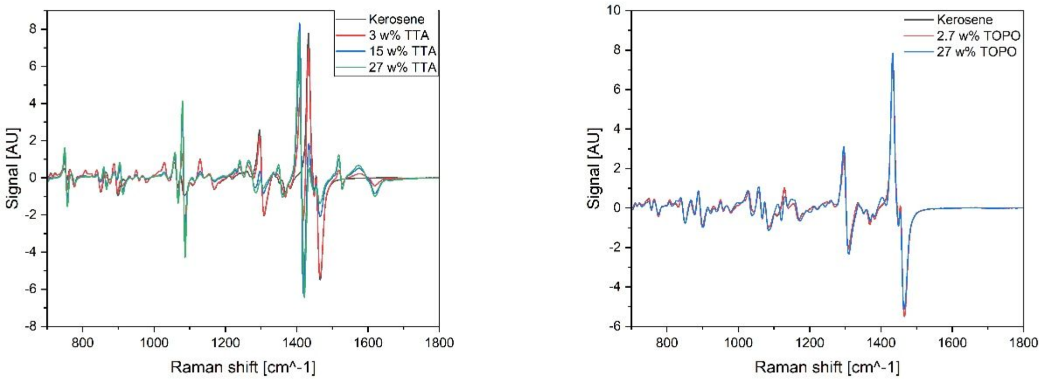

Figure S15: Preprocessed Raman Spectra of TTA in kerosene (left) and TOPO in kerosene (right).

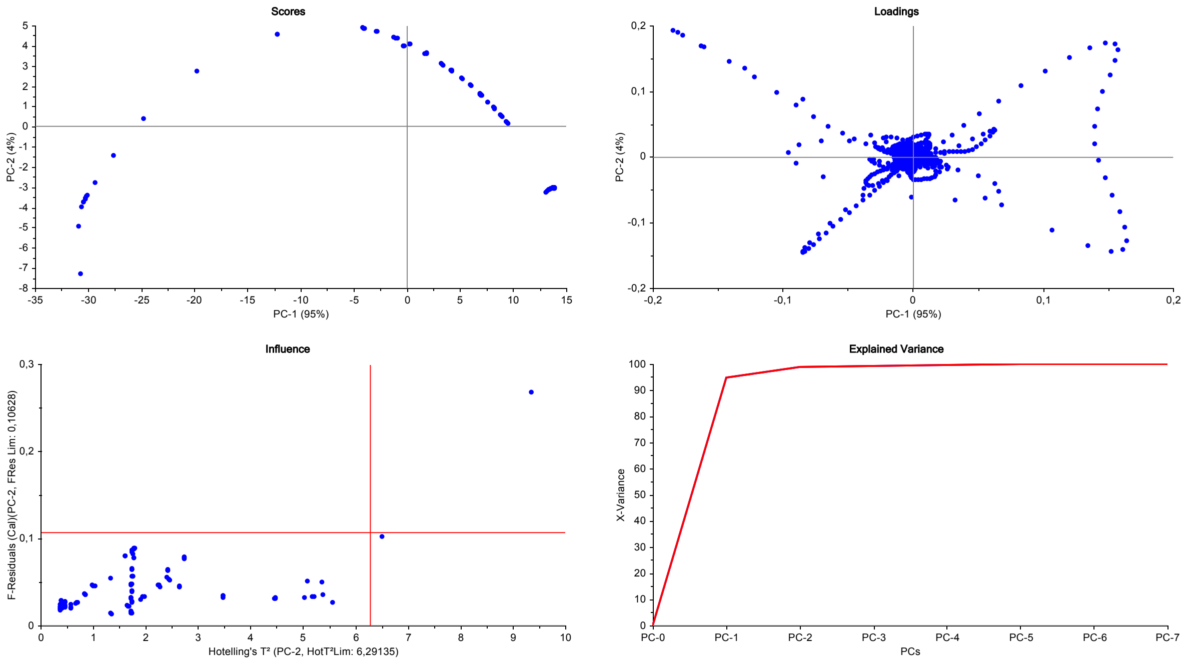

Figure S16: Summarized overview of the PCA after preprocessing.

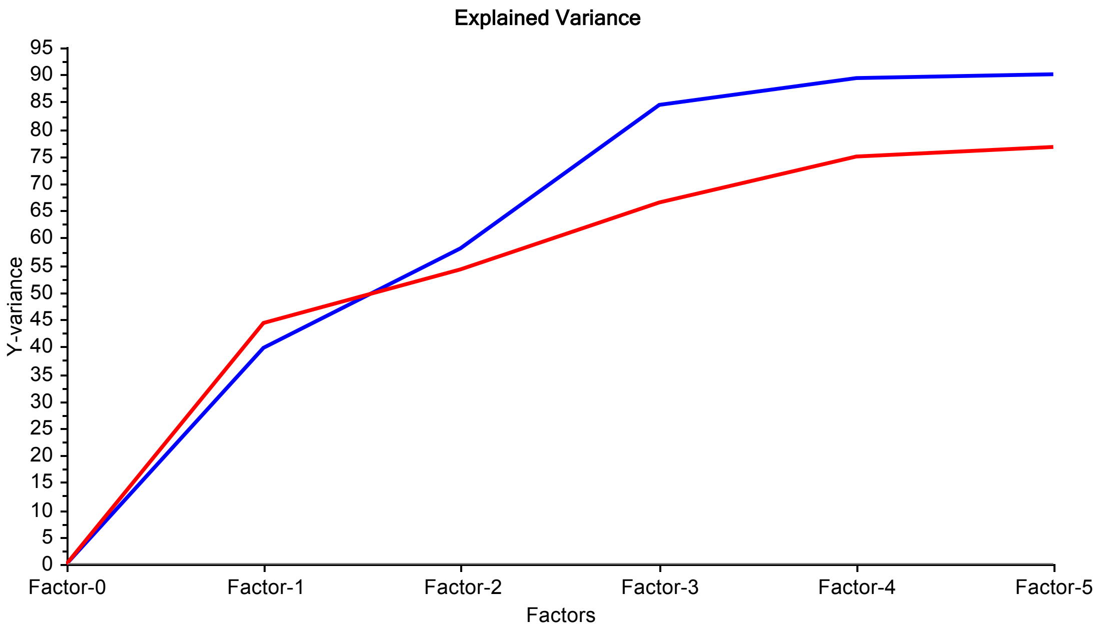

Figure S17: Explained variance by the regression factors of the PLS analysis.

3.2 Determination of the Degree of Saponification

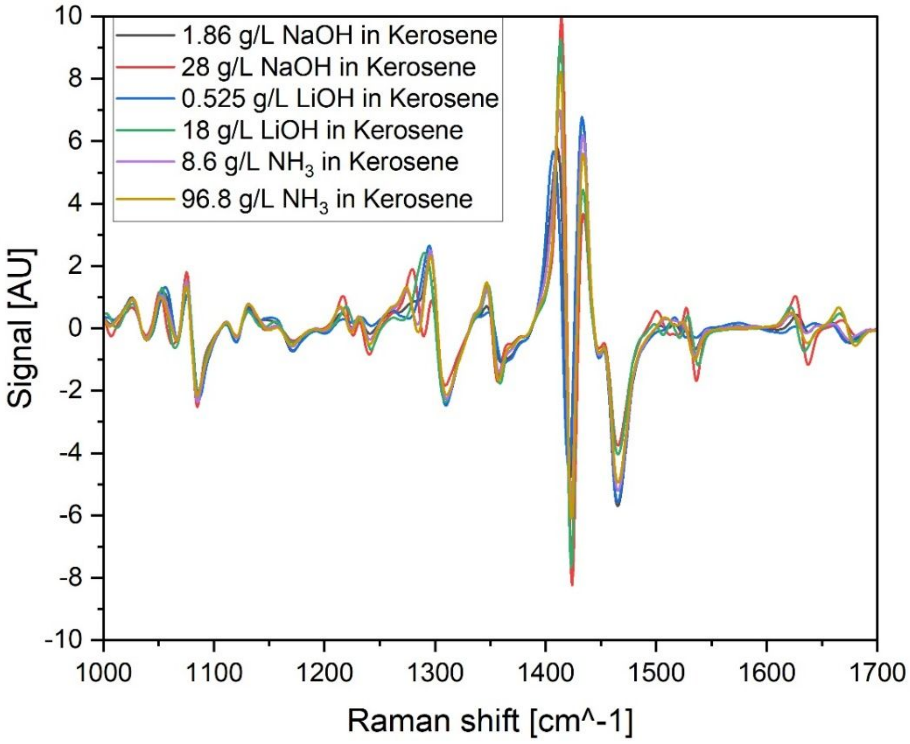

Figure S18: Preprocessed spectra of saponificated organic phase with NaOH, LiOH and NH_3.

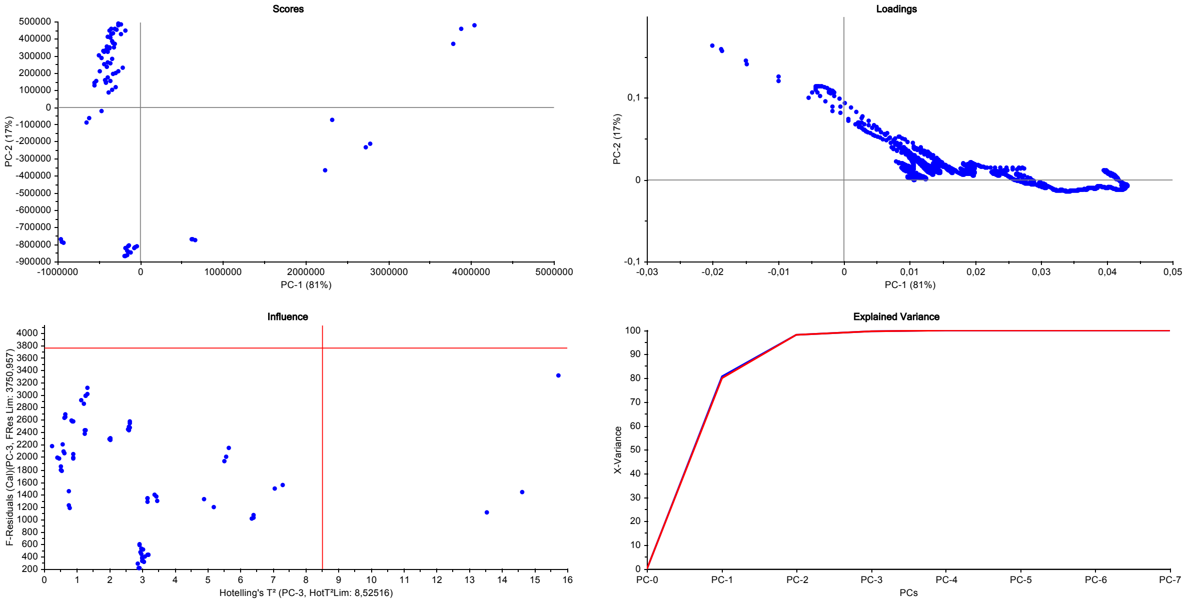

Figure S19: Summarized overview of the PCA before preprocessing and outlier-removal.

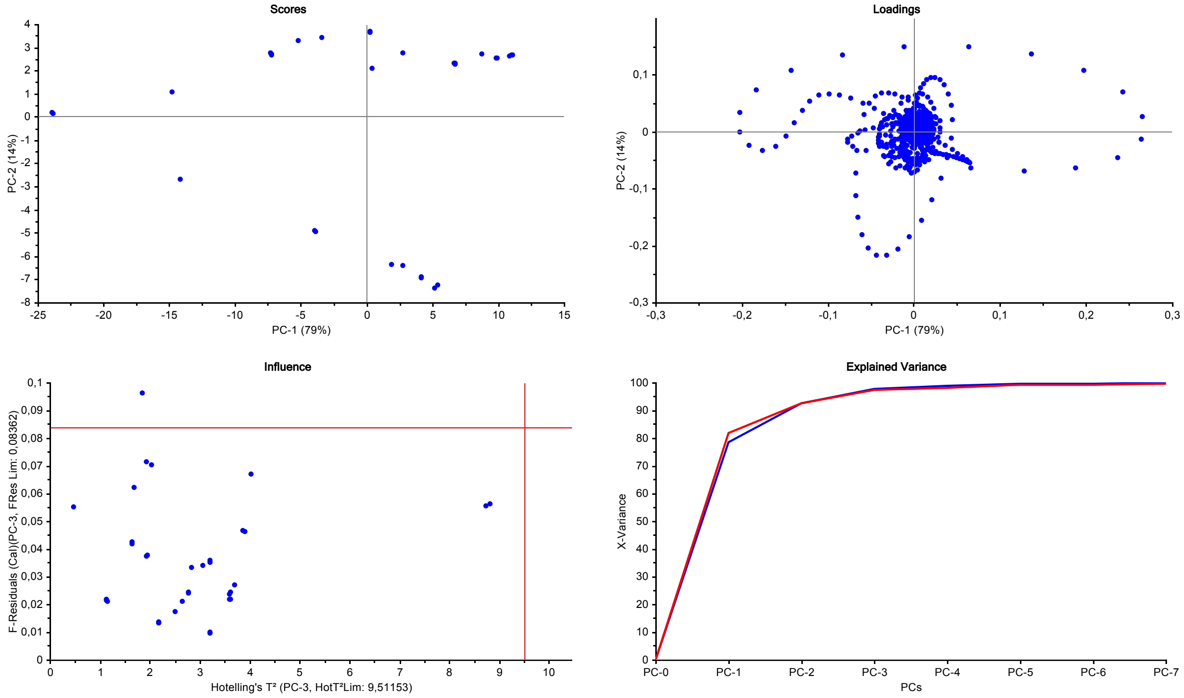

Figure S20: Summarized overview of the PCA after preprocessing.

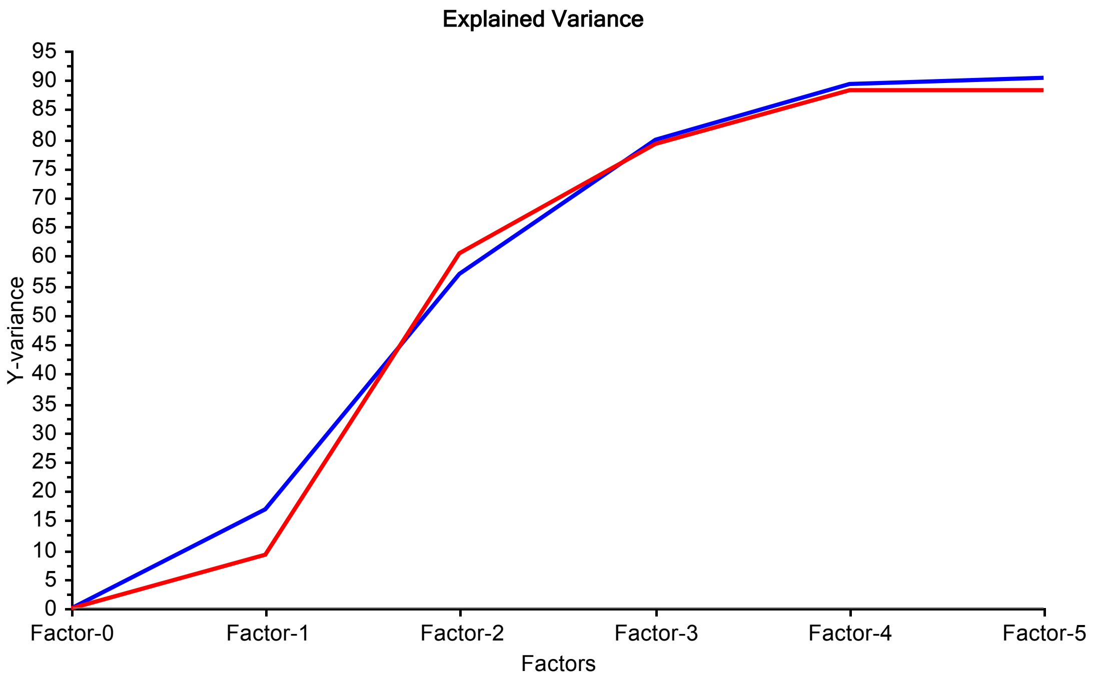

Figure S21: Explained variance by the regression factors of the PLS analysis.

3.3 Total metal concentration

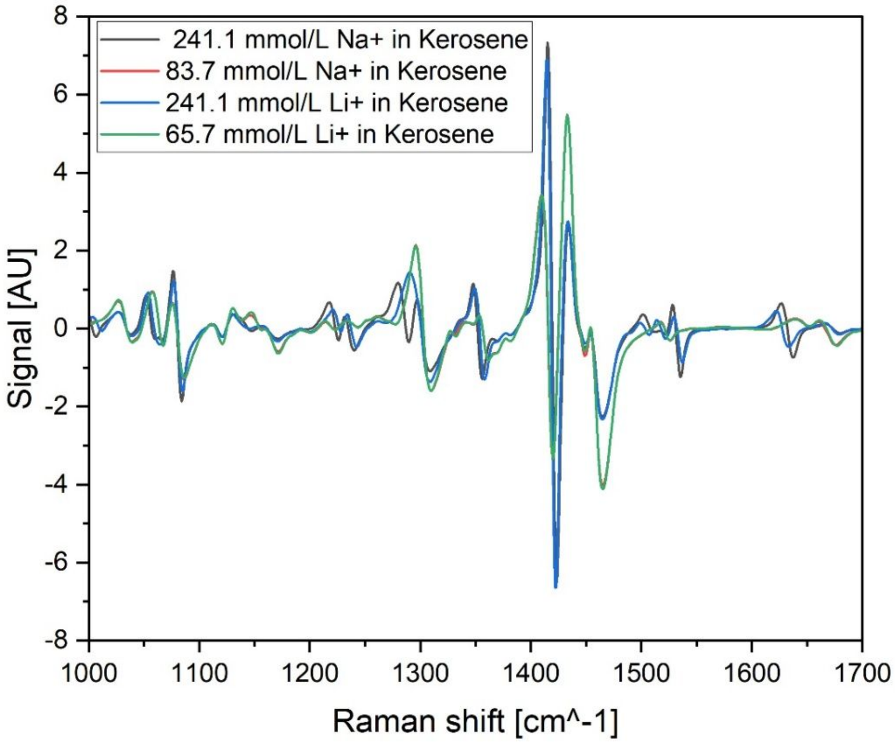

Figure S22: Preprocessed Raman spectra of organic phase with Na and Li complexes.

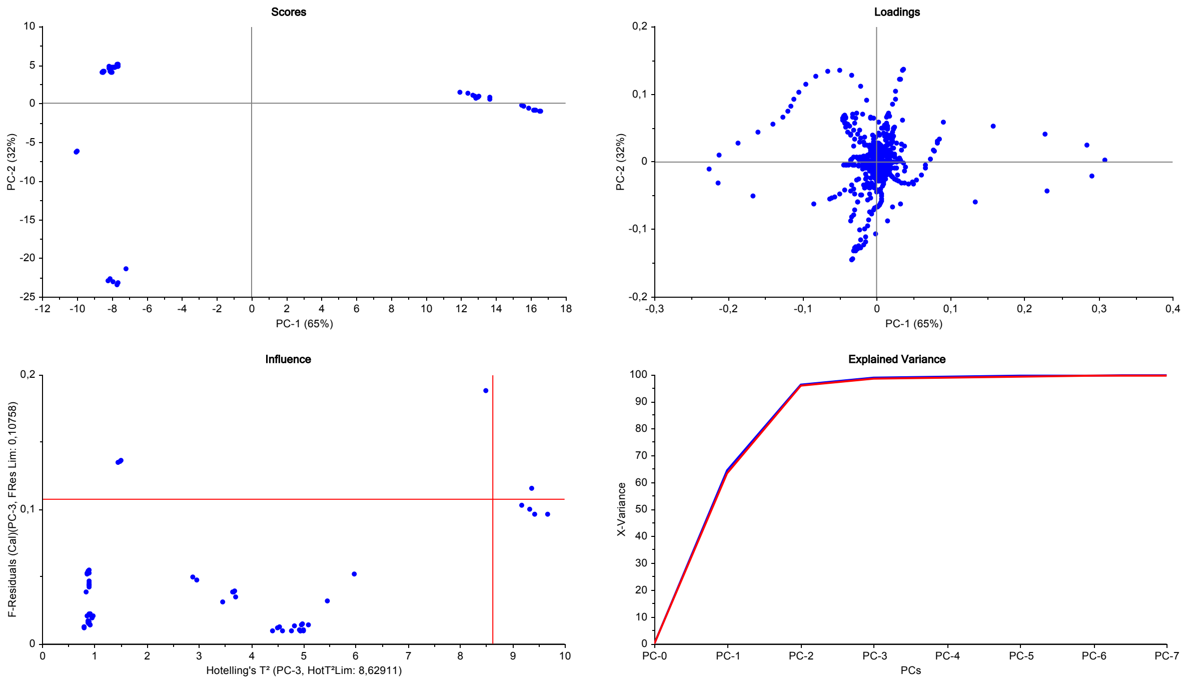

Figure S23: Summarized overview of the PCA after preprocessing.

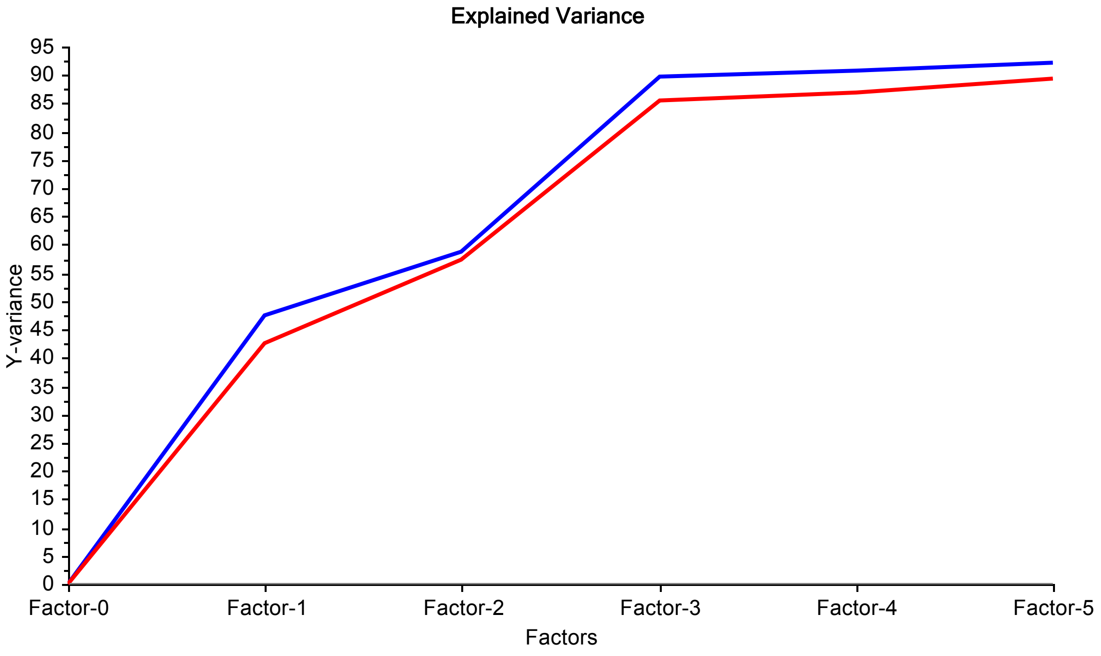

Figure S24: Explained variance by the regression factors of the PLS analysis.

Section 4: MCR-ALS model validation via regression parity diagrams

4.1 TTA and TOPO measurements

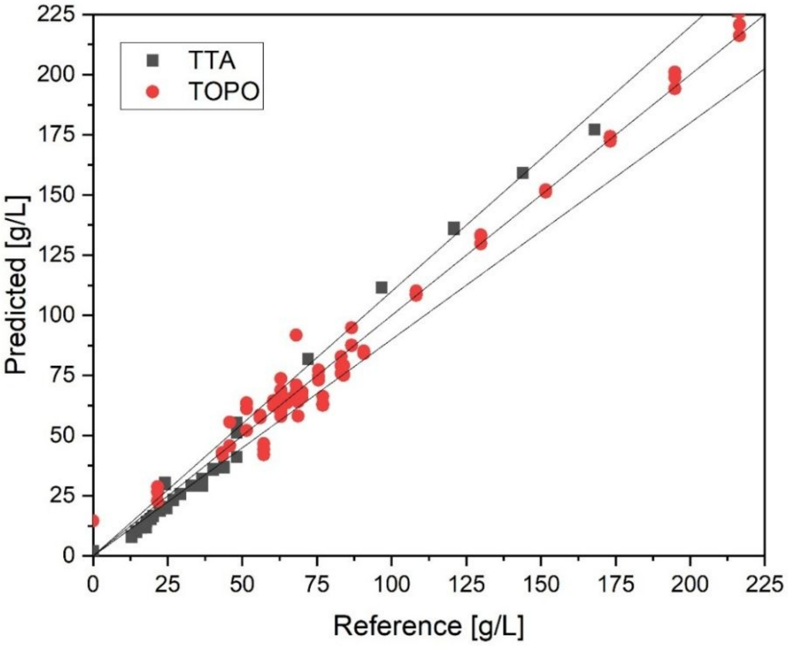

Figure S25: MCR-ALS regression parity diagram for the determination of TTA and TOPO concentration from FT-IR spectra.

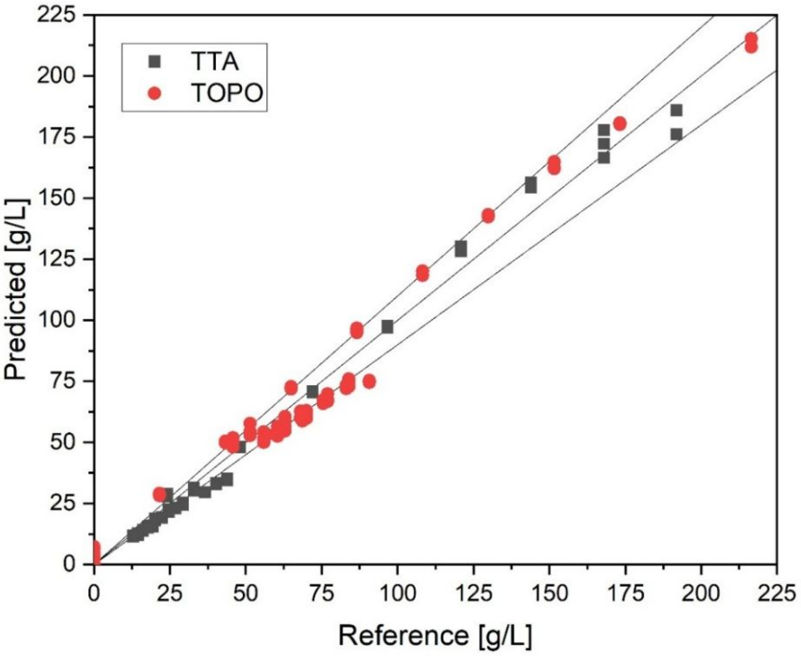

Figure S26: MCR-ALS regression parity diagram for the determination of TTA and TOPO concentration from Raman spectra.

4.2 Determination of the Degree of Saponification

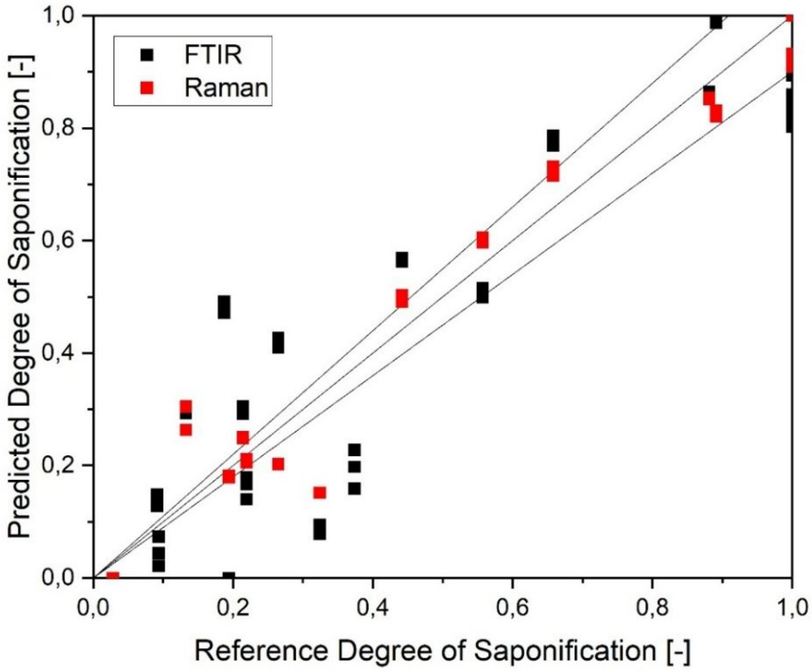

Figure S27: MCR-ALS regression parity diagram for the determination of degree of saponification from FT-IR and Raman spectra.

4.3 Total metal concentration

4.3.1 Total metal concentration determined with FT-IR

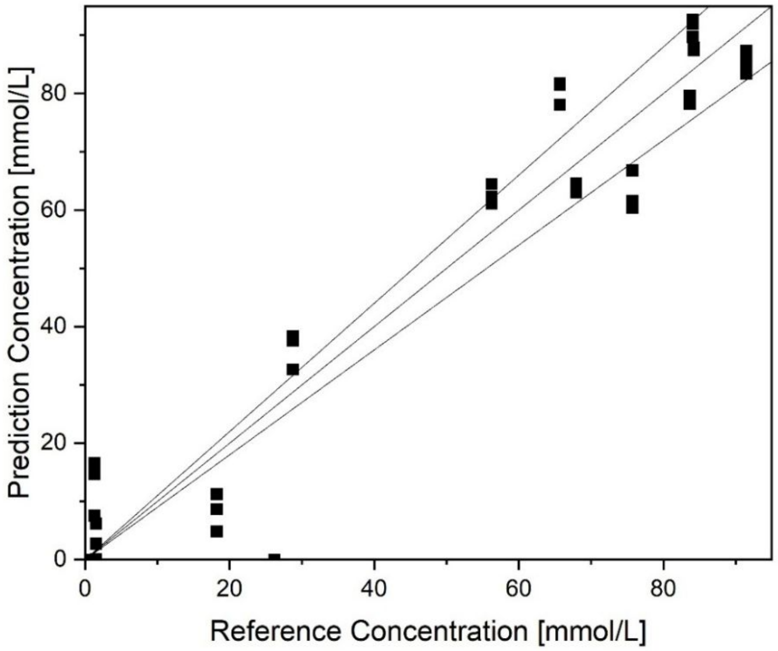

Figure S28: MCR-ALS regression parity diagram for the determination of metal ion complex concentration from FT-IR spectra for low concentration region.

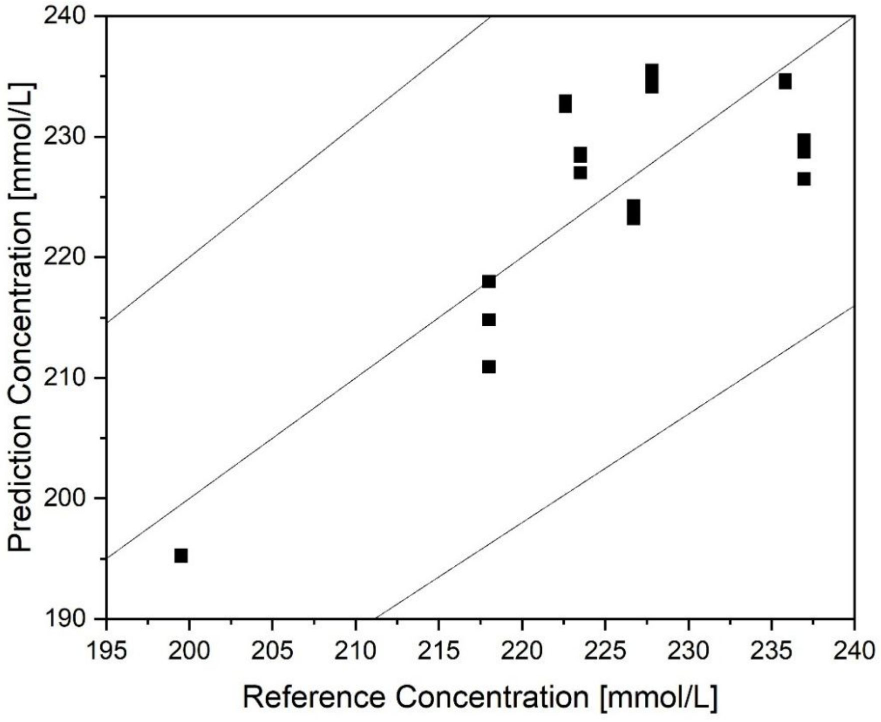

Figure S29: MCR-ALS regression parity diagram for the determination of metal ion complex concentration from FT-IR spectra for high concentration region.

4.3.2 Total metal concentration determined with Raman

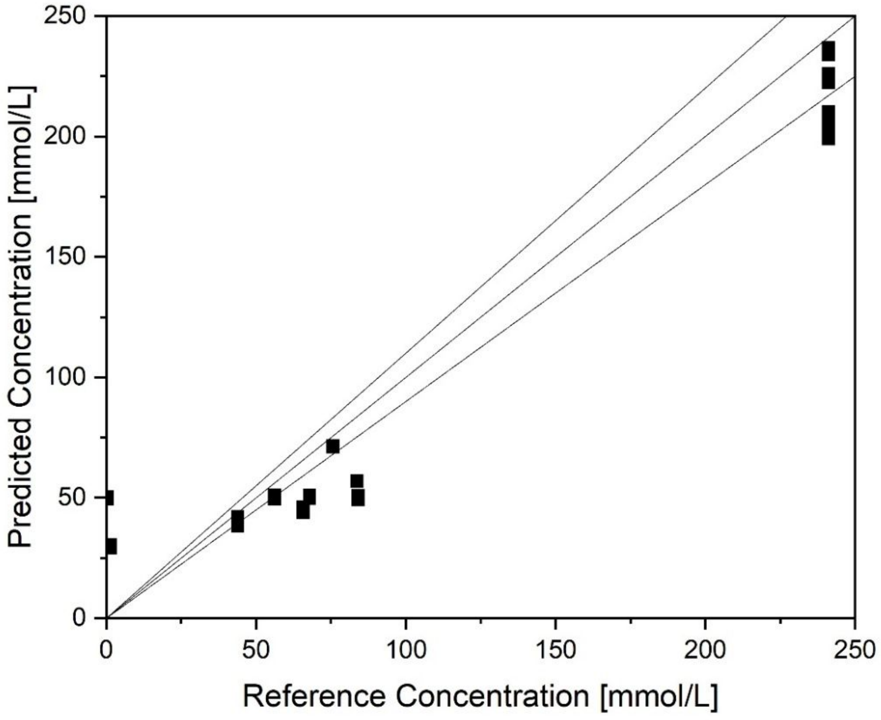

Figure S30: MCR-ALS regression parity diagram for the determination of metal ion complex concentration from Raman spectra
